# Fear of Missing Out’s (FoMO) relationship with moral judgment and behavior

**DOI:** 10.1371/journal.pone.0312724

**Published:** 2024-11-07

**Authors:** Paul C. McKee, Ithika Senthilnathan, Christopher J. Budnick, Marie-Abèle Bind, Imad Antonios, Walter Sinnott-Armstrong

**Affiliations:** 1 Department of Psychology & Neuroscience, Duke University, Durham, North Carolina, United States of America; 2 Center for Cognitive Neuroscience, Duke University, Durham, North Carolina, United States of America; 3 Duke Institute for Brain Sciences, Duke University, Durham, North Carolina, United States of America; 4 Department of Psychology, Southern Connecticut State University, New Haven, Connecticut, United States of America; 5 Biostatistics Center, Massachusetts General Hospital, Boston, Massachusetts, United States of America; 6 Department of Medicine, Harvard Medical School, Boston, Massachusetts, United States of America; 7 Department of Computer Science, Southern Connecticut State University, New Haven, Connecticut, United States of America; 8 Department of Philosophy, Duke University, Durham, North Carolina, United States of America; 9 Law School, Duke University, Durham, North Carolina, United States of America; 10 Kenan Institute for Ethics, Duke University, Durham, North Carolina, United States of America; Aalborg University, DENMARK

## Abstract

Across three online studies, we examined the relationship between the Fear of Missing Out (FoMO) and moral cognition and behavior. Study 1 (N = 283) examined whether FoMO influenced moral awareness, judgments, and recalled and predicted behavior of first-person moral violations in either higher or lower social settings. Study 2 (N = 821) examined these relationships in third-person judgments with varying agent identities in relation to the participant (agent = stranger, friend, or someone disliked). Study 3 (N = 604) examined the influence of recalling activities either engaged in or missed out on these relationships. Using the Rubin Causal Model, we created hypothetical randomized experiments from our real-world randomized experimental data with treatment conditions for lower or higher FoMO (median split), matched for relevant covariates, and compared differences in FoMO groups on moral awareness, judgments, and several other behavioral outcomes. Using a randomization-based approach, we examined these relationships with Fisher Tests and computed 95% Fisherian intervals for constant treatment effects consistent with the matched data and the hypothetical FoMO intervention. All three studies provide evidence that FoMO is robustly related to giving less severe judgments of moral violations. Moreover, those with higher FoMO were found to report a greater likelihood of committing moral violations in the past, knowing people who have committed moral violations in the past, being more likely to commit them in the future, and knowing people who are likely to commit moral violations in the future.

## Introduction

Individuals face numerous scenarios daily involving moral content. Yet, situational and individual differences can significantly impact moral information processing and resulting judgments. One such individual difference, the Fear of Missing Out (FoMO)–apprehension that others may have rewarding experiences from which one is absent [1]–demonstrates high individual variability and might account for differences in moral cognition. Indeed, those with higher levels of FoMO are more likely to engage in behaviors that could be considered morally questionable, such as texting while driving [2]–thereby endangering others–or participating in academic misconduct, like plagiarism and classroom incivility [3]. Despite these implications, research has yet to examine FoMO’s influence on moral cognition. Addressing this gap, this paper reports findings from three large-sample studies examining the relationships between FoMO and moral awareness, judgments, and behavior recall and prediction using causal modeling, traditional statistical, and machine learning approaches.

### Fear of Missing Out

FoMO’s social distress is widespread. Only 13% of individuals report never experiencing FoMO [4], with the highest prevalence observed in those 18 to 34 years old [5, 6]. Currently, most work focuses on young adults and social media [7], with relatively less on FoMO’s influence in other areas like increasing workplace burnout [8]. More specifically, no studies have examined its effect on moral cognition.

FoMO is known to be associated with lower life satisfaction, poorer mood, lower psychological need satisfaction, anxiety, and self-esteem [1, 9]. However, FoMO exists not just as affect or cognition (i.e., anxiety, stress, rumination). FoMO also results in behaviors–beyond increased social media and phone use—with both realized and potential consequences for others. For example, higher FoMO students are more likely to use phones during class or while driving and engage in more maladaptive behavior (i.e., illegal substance use, alcohol abuse, giving away prescription drugs, stealing, and academic misconduct [3]. Those with higher levels of FoMO exhibit more maladaptive behavior, suggesting some type of self-regulation failure. This raises the question of whether moral information is processed differently between higher and lower FoMO individuals. Importantly, we are not conflating maladaptive behavior with immorality; rather, we acknowledge that considerable overlap exists among such behaviors.

### Moral awareness

The first step in processing moral information is recognizing that an action or situation is morally relevant. Reynolds [10] defined moral awareness as one’s “determination that a situation contains moral content and legitimately can be considered from a moral point of view.” The issue-contingent model [11] suggests six factors shape moral awareness: 1) the magnitude of the consequences derived from behaviors engaged in, 2) social consensus towards the situation, 3) probability of expected outcomes, 4) temporal immediacy of behavior and expected consequence, 5) proximity of moral situation and agents, and 6) concentration of the expected effect of behavior.

Relevant to social consensus (factor 2), Butterfield and colleagues [12] argue the most important contributor to moral issue identification is the social context surrounding an individual in any given situation. When individuals perceive social consensus that behavior is ethically problematic, they are more likely to exhibit moral awareness. Alternatively, if social consensus indicates behavior is not an ethical concern, individuals will likely not experience awareness of that situation as involving moral components. Moreover, the social setting affects perceptions of available and achievable resources. The conservation of resources (COR) theory [13] addresses the motivations that prompt individuals to maintain their current resources while pursuing new ones. COR describes the development of psychological stress when there is a threat of resource loss, actual loss, or failure to gain resources after expending resources. Resources need not be tangible, such as goods, money, or services. Rather, resources can be social, such as affection, love, information, or status [14].

Others’ rewarding experiences are some of the social resources that Hobfoll’s COR and Foa and Foa’s social resource theories address, so FoMO can then be understood as anxiety about missing out on this particular social resource. As anxiety is associated with hypervigilance [15], it is likely that those high in FoMO are especially sensitive to social resource threats (real and anticipated). This hypervigilance can lead them to focus on non-moral considerations and/or overlook (or reduce) awareness of moral content in some situations, resulting in an inability to accurately judge others’ or effectively judge one’s morality [16, 17].

### Moral judgment

Once aware of the moral nature of a situation, individuals quickly and effortlessly render judgments regarding responses or behaviors within that context [18]. Rapid moral reasoning is important in social contexts where people try to influence each other and gain consensus [19]. Although moral rules are sometimes thought to be rigid and universal [20], moral decision-making and moral judgments are highly sensitive to situational, cultural, and individual differences [21–24]. Others find that post-hoc reasoning often justifies behavior [25, 26] and that moral reasoning and judgments may be biased to defend against harmful ideas and maintain social relationships [27].

### The present studies

The present research explores the impact of FoMO on moral awareness, judgments, and behaviors through three studies using causal modeling, traditional statistical analysis, and machine learning approaches. In Study 1, we investigate whether FoMO affects moral judgments of first-person moral violations in different social settings (highly social: presence of friends, lowly social: absence of friends). In Study 2, we replicate and extend upon the initial findings, examining third-person judgments with varying agent identities in relation to the participant (stranger, friend, or someone disliked). In Study 3, we examine the influence of recalling activities either participated in or missed (meant to induce FoMO) on moral judgments. Across these studies, we gathered individual difference measures (e.g., personality, need for cognition, religious fundamentalism, etc.) known to be associated with FoMO or moral cognition, or that were anticipated to exhibit differences between those with higher and lower levels of FoMO. This comprehensive array of measures was crucial to account for as many relevant baseline differences in people as possible for our causal modeling and inferences.

## Study 1

In the first study, we sought initial evidence that FoMO is associated with moral judgments. A recent study [3] highlights FoMO’s relationship with maladaptive behaviors. Specifically, that study reports findings of elevated levels of illegal behavior, drug and alcohol consumption, and academic misconduct in college students with higher FoMO levels. Moreover, even when considering combinations of other relevant demographic variables (i.e., sex, living situation, socioeconomic status) in supervised machine learning models, FoMO held, by far, the highest predictive value for all maladaptive behaviors.

Not all maladaptive behaviors are immoral (i.e., video game addiction, not bathing). Still, all immoral behaviors (i.e., stealing, infidelity, murder) are maladaptive, at least to the extent that they disrupt cooperation, as morality is argued to have evolved to facilitate cooperation [28–30]. Therefore, an important consideration is whether prior findings regarding illegal behaviors [3] extend to explicitly immoral behaviors.

High FoMO individuals may be more likely to commit moral violations to remain engaged with their social circle, staying aware and participating in everything possible to alleviate the chronic anxiety that they would otherwise face. Furthermore, engaging with people who exhibit such moral violations may alter previous moral intuitions through repeated exposure, or judgments may be shifted purposefully to avoid negative attributions to oneself and others. Although the team was split regarding the direction of the influence of FoMO on moral awareness, we ultimately hypothesized that higher FoMO would predict lower moral awareness levels. We also hypothesized that higher levels of FoMO and highly social settings would uniquely associate with less severe moral judgments.

Regarding our machine learning approaches, we proceeded with the following research questions for all studies: If FoMO is found to have a relationship with moral judgments, how much predictive ability does FoMO have in predicting moral judgments and how much predictive ability do moral awareness, judgments, and behaviors have in predicting FoMO? If there is meaningful predictive performance for either of the above, how much predictive weight does each feature have compared to one another? In other words, which features are most important in predicting the outcome variables of interest?

### Method

#### Participants

We recruited a panel of participants (N = 283) representative of the United States of America through Qualtrics (see Table 1). All participants gave informed consent electronically online. Institutional Review Board (IRB) approval was obtained from Southern Connecticut State University.

**Table 1:** Study participants.

	Study 1 (N = 283)	Study 2 (N = 821)	Study 3 (N = 604)
Age	48.00 (17.95)	47.00 (18.33)	49.00 (17.85)
Female (%)	53.36	55.66	54.80
White	59.36	62.61	65.23
Black/African American	13.78	11.69	11.42
Hispanic or Latino	17.31	17.05	14.90
Asian	5.65	5.24	5.46
American Indian/Alaska Native	0.71	0.24	0.66
Native Hawaiian/Pacific Islander	0.00	0.12	0.17
Two or more races	3.18	3.05	2.15

Note. Age is median with standard deviation in parentheses. All other demographics are represented as a percent. Samples were meant to be diverse while approximating national representation in the United States for age, sex, and race.

#### Procedures and materials

Participants completed demographic, FoMO, personality, social media usage, and need for cognition measures (see Table 2). They read 14 vignettes describing moral violations in one of two randomly assigned conditions. The highly social condition consisted of highly social vignettes (e.g., supporting characters in the vignettes were the participant’s “best friends” and “closest friends”). The low social condition consisted of vignettes that were not as social (e.g., absence of best or closest friends). After each vignette, participants completed a moral awareness measure, examining how readily they identified the vignette content as morally relevant. Next, participants reported a moral judgment for each vignette. After giving a moral judgment, participants responded to questions about personal and social recall (i.e., have they or anyone they know done something like this?) and personal and social prediction (i.e., how likely is it that you or someone you know would do something like this?). Upon completing all the vignettes, participants answered questions about FoMO (a second time), religious fundamentalism, and political identity. Although FoMO has been primarily viewed and validated as a trait variable, having FoMO measures before and after the experimental trial served two purposes: 1) the double administrations acted as a validation tool to check the accuracy of participant engagement and effort, and 2) provided exploratory data for the possibility that FoMO may also function as a state variable.

**Table 2:** Measures used in studies.

Variable	Number of Items	Measure	Sample Item	Scale Anchors	Reliability	Study
Fear of Missing Out	10	[1]	“I fear my friends have more rewarding experiences than me.”	1 = Not at all true of me—5 = Extremely true of me	.87	1–3*
Imagination	4	[31]	“Have a vivid imagination.”	1 = Very inaccurate—5 = Very accurate	.62	1–3
Conscientiousness	4	[31]	“Get chores done right away.”	1 = Very inaccurate—5 = Very accurate	.51	1–3
Extraversion	4	[31]	“Talk to a lot of different people at parties.”	1 = Very inaccurate—5 = Very accurate	.75	1–3
Agreeableness	4	[31]	“Sympathize with others’ feelings.”	1 = Very inaccurate—5 = Very accurate	.75	1–3
Neuroticism	4	[31]	“Get upset easily.”	1 = Very inaccurate—5 = Very accurate	.68	1–3
Social Media Use	10	Adapted from [32]	“Social media is a part of my everyday activity.”	1 = Strongly disagree—5 = Strongly agree	.93	1
Need for Cognition	18	[33]	“I prefer complex to simple problems.”	1 = Extremely uncharacteristic of me—5 = Extremely characteristic of me	.90	1–3
Religious Fundamentalism	12	[34]	“The fundamentals of God’s religion should never be tampered with, or compromised with others’ beliefs.”	1 = Very strongly disagree—9 = Very strongly agree	.80	1–3
Political Leaning	1	[35]	-	1 = Strongly liberal—7 = Strongly conservative	-	1–3
Moral Awareness	3	Adapted from [10]	“This situation could be described as a moral issue.”	1 = Strongly disagree—7 = Strongly agree	.78	1–3
Moral Judgment	1	-	“How would you classify the behavior in the scenario?”	-100 = Morally wrong—100 = Morally right	-	1–3
Personal Recall	1	-	“Have you ever engaged in a behavior similar to this scenario in real life?”	1 = Absolutely no—7 = Absolutely yes	-	1–3
Social Recall	1	-	“Do you know of anyone personally that has engaged in a behavior similar to this scenario in real life?”	1 = Absolutely no—7 = Absolutely yes	-	1–3
Personal Prediction	1	-	“How likely is it that you would engage in a behavior similar to this scenario in real life?”	1 = Absolutely would not happen—7 = Absolutely would happen	-	1–3
Social Prediction	1	-	“How likely is it that someone you know personally would engage in a behavior similar to this scenario in real life?”	1 = Absolutely would not happen—7 = Absolutely would happen	-	1–3

Note

* The FoMO measure was completed twice for each of these studies. Cronbach alphas are reported from Study 1, “-” where not relevant.

#### Measures

*Vignettes*. Vignettes described a moral violation in a high or low social setting. Vignettes were validated, and results are reported in Supplemental Information S1 Text. Examples for both conditions are as follows (words in bold are for emphasis and were not shown as such to participants):

*Highly social*. “You’re working on the night of the Super Bowl. You were supposed to get out in time to watch it with **all your closest friends who are waiting for you**, but your relief is running late. These have been **your best friends** since you were a kid. By the time they finally arrive, it’s almost half-time. You go 100 mph on the highway, so you don’t miss the halftime show with **your friends**.” Please note that the bold font is just used here to emphasis the difference between the two primary conditions, it was not presented to participants this way.

*Non-social*. “You’re working on the night of the Super Bowl. You were supposed to get out in time to go home and watch it alone, but your relief is running late. By the time your relief finally arrives, it’s almost half-time. You go 100mph on the highway, so you don’t miss the halftime show.”

#### Statistical analysis

For each study, to estimate the causal effects between FoMO and outcomes of interest, we used the Rubin Causal Model (RCM) [36]. Among others, Bind and Rubin [37] call for the creation of hypothetical experiments randomizing higher (experimental treatment) vs. lower FoMO (control). To do this, we classified participants in each study using a binary FoMO “treatment”: higher FoMO or lower FoMO (split on the median for each study). Participants at or below the median were assigned to lower FoMO, while participants above the median were assigned to the higher FoMO group. Although relatively uncommon in psychology, this approach has recently been used to estimate combat exposure’s causal effects on drinking behaviors and subjective well-being [38].

W_i_ serves as the exposure indicator (i.e., 1 if unit i is in the experimental treatment arm, 0 if otherwise in the control arm). For hypothetical experiments, treatment is assumed to be randomly assigned to the experimental treatment arm (W_i_ = 1) or the control arm (W_i_ = 0). We assume that these hypothetical randomized experiments satisfy the Stable Unit Treatment Value Assumption (SUTVA) [39] and that, given background covariates, the exposure assignment mechanism is unconfounded [40]. SUTVA reduces the potential outcomes for each participant to just two: Y_i_(W_i_ = 1), the level of moral judgment (or other outcomes) for participant i had participant i been exposed to higher FoMO (i.e., W_i_ = 1) and Y_i_(W_i_ = 0) the level of moral judgment (or other outcomes) that occurs for participant i had participant i been exposed to lower FoMO (i.e., if W_i_ = 0). We denote the observed level of the outcome moral judgment for participant i by Y_i_^obs^, which equals Yi(Wi = 1) if Wi = 1 or Y_i_(W_i_ = 0) if W_i_ = 0.

These hypothetical randomized experiments are constructed without the observed outcome of interest to replicate true randomized experiments’ ideal condition assumptions and avoid potential p-hacking [41]. Rubin [42] also suggests that the treatment assignment mechanism can be unconfounded sufficiently well by a matched-sampling approach. For each of these studies, we constructed two equal-sized matched groups with a caliper of various proportions of the standard deviation for estimated propensity scores. A propensity score is the probability of one of our units (i.e., a participant) being assigned to one of our hypothetical treatment arms (higher or lower FoMO) given a set of measured background covariates. The idea is that matching propensity scores reduce baseline differences between groups by making them as similar as possible with respect to these observed background covariates. Eliminating baseline differences is crucial to draw causal inferences. Any covariate standardized mean difference between groups outside the [-1.96; 1.96] interval in the Love plot [43] was considered unbalanced–anything within is considered sufficiently balanced.

After matching, we conducted Fisher tests [44] comparing our binary FoMO treatment groups on each dependent variable using the observed mean difference’s absolute value as the test statistic. We ran 100,000 permutations for our tests to approximate Fisher-exact p-values. Most analyses of randomized experiments default to report asymptotic p-values instead of relying on the actual randomization procedure that led to the observed data, and Fisher and asymptotic p-values can differ dramatically [45]. For the Fisher tests, we also calculated Fisherian Intervals (FI) instead of standard confidence intervals (CI) for similar reasons. Additional exploratory analyses examined any relationships between FoMO and personal and social recall, and personal and social prediction.

We also ran general additive models (GAM) to estimate conditional associations between FoMO (as a continuous exposure) on our dependent variables using our unmatched data. All continuous variables were entered first as non-linear with cubic regression splines. If found to be linear (effective degrees of freedom less than two), those variables were then reentered into the model as such. After rerunning the model, all variables that did not explain variance were removed. Predictor variables with p-values less than 0.1 remained until the final model was settled upon. This two-step process repeated until continuous variables settled on linearity or not, and only predictors that explained variance remained. Experimental conditions and FoMO remained in the model regardless of their p-values. All GAMs report asymptotic p-values and standard CIs.

**Fig 1:**
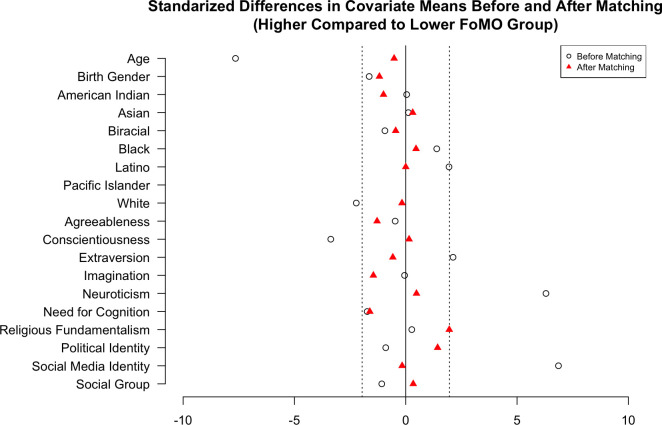
Study 1 covariate Love plot. Fig 1 shows the standardized differences in covariate means before (white circle) and after (red triangle) matching.

All the measures outlined above were included in Study 1. For our RCM treatment, the median FoMO value was 1.70. Before matching, the unbalanced covariates between groups (i.e., baseline differences between lower and higher FoMO) were age, the proportion of white people, conscientiousness, extraversion, neuroticism, and social media identity. After matching with a caliper of 0.1 (standard deviation of the estimated propensity scores), we could not observe any evidence of imbalance for these variables. Please see Fig 1 for the Love plot. This matching decreased the sample size from 283 to 140 participants.

For our GAM modeling, we noticed a few outliers and included only participants with moral awareness and judgment scores greater than 2.5 and less than 50, respectively. Thus, our Fisher tests used 140 matched participants, while our GAM models used 272 participants. All analyses for all studies, including matching, used a set random seed for reproducibility.

#### Machine learning

We used the unmatched dataset for our machine learning approach to maintain the full variability between participants and leverage as much data as possible. In line with our research questions, our two goals were to predict moral judgment and FoMO (as continuous outcomes). The regression algorithms used include Ridge Regression (Ridge), Support Vector Regression (SVR), Random Forest (RF) Regressor, and XGBoost, whose implementations are provided by the Python machine learning library scikit-learn. For an in-depth examination of these models, please see [46], as it is out of the scope of this paper. The Ridge model was trialed using several solvers such as ‘svd’, ‘cholesky’, ‘lsqr’, and ‘sag’, coupled with several regularization strengths (alpha values from 1e-5 to 100). Additionally, configurations both inclusive and exclusive of an intercept term were explored. The SVR model was assessed by searching through multiple penalty parameter C and coefficient gamma values. Random Forest had maximum tree depth, and minimum sample threshold hyperparameters searched through. Lastly, for XGBoost the number of boosting rounds, the tree’s maximum depth, and step size shrinkage were adjusted. Although not exhaustively searched, the results from the optimal configuration for all of these models are reported.

Model performance for both outcomes was evaluated by two distinct metrics, R-squared (*R*^2^) and Mean Absolute Error (MAE). *R*^2^ represents the proportion of the dependent variable’s variance attributable to the independent variables, or predictive features, with a possible perfect score of 1 (i.e., the further from 0, the better). MAE is the mean value of the absolute differences between predicted and actual outcomes, with the ideal MAE being minimized (i.e., the closer to 0, the better). It is important to note that as an absolute error metric, MAE is scale-dependent. Thus, an interpretation of its magnitude should consider the MAE’s value relative to the range of the response variable.

Feature importance was deduced from the Random Forest Regressor model. For each predicted outcome (moral judgment or FoMO), the contribution of each feature in predicting the outcome was represented through bar plots. These plots show the mean decrease in node impurity for each feature and the associated standard deviations across trees.

To make results more comparable across studies for a given response (FoMO or moral judgment), only features common across all studies were included as predictors (e.g., no experimental manipulations that varied across studies). The features used in predicting FoMO are age, birth gender, political identity, extraversion, agreeableness, conscientiousness, neuroticism, imagination, religious fundamentalism, need for cognition, moral awareness, moral judgment, personal recall, social recall, personal prediction, and social prediction. When moral judgment was the dependent variable, moral judgment as a predictor was replaced by FoMO.

### Results

#### Rubin causal model

Our Fisher tests comparing our FoMO treatment arms provided no evidence of gross differences in outcomes. However, the estimated average causal effects point in the direction of the hypotheses. See Table 3 for full results.

**Table 3:** Study 1 Fisher test results.

	Higher FoMO (N = 70)	Lower FoMO (N = 70)	Estimated Average Causal Effect	Fisher p-value	95% Fisherian Intervals
Moral Awareness	5.19 (1.05)	5.45 (0.94)	-0.25	.1354	[-0.63, 0.13]
Moral Judgment	-47.95 (38.12)	-56.02 (30.08)	8.06	.1680	[-5.07, 21.24]
Personal Recall	2.28 (1.13)	2.00 (1.17)	0.28	.1515	[-0.16, 0.72]
Social Recall	3.25 (1.46)	3.14 (1.69)	0.11	.6868	[-0.49, 0.70]
Personal Prediction	2.14 (2.08)	1.87 (0.92)	0.27	.1092	[-0.10, 0.65]
Social Prediction	3.23 (1.50)	3.08 (1.61)	0.15	.5724	[-0.44, 0.74]

Note. Means are presented for all outcome variables for both treatment arms, with standard deviations in parentheses. Fisher p-values are based on 100,000 permutations as are the Fisherian Intervals.

#### Generalized additive model

*Moral awareness*. The model accounted for 29.2% of the deviance (variance: *adjusted R*^*2*^ = .26). The parametric terms revealed several significant predictors of moral awareness. Age was positively associated with moral awareness, such that as age increased, so did levels of moral awareness (*b* = 0.02, *SE* = 0.01, *t*(260) = 4.73, *p* < .001). Additionally, participants identifying as Black (compared to White) were associated with significantly lower moral awareness (*b* = -0.52, *SE* = 0.15, *t*(260) = -3.43, *p* = .001). Furthermore, conscientiousness was also a significant predictor, with higher conscientiousness associated with greater moral awareness (*b* = 0.17, *SE* = 0.07, *t*(260) = 2.39, *p* = .018). Approaching traditional statistical significance, being in the highly social condition and moral awareness were found to have potentially been associated (*b* = -0.18, *SE* = 0.10, *t*(260) = -1.81, *p* = .071).

Regarding non-linear relationships we could not reject the null when testing for the smoothed effect of FoMO on moral awareness (*edf* = 3.25, *F* = 1.58, *p* = .018). However, the smooth term for social media identity was significant, revealing a complex, non-linear relationship with moral awareness (*edf* = 4.61, *F* = 4.35, *p* < .001). Interested readers are directed to Supplemental Information S1 Text for plots of all non-linear relationships throughout all three studies, as only FoMO and experimental variables (e.g., social group) that show significant non-linear relationships will be discussed for brevity.

*Moral judgment*. The model explained 30.4% of the deviance (variance: *adjusted R*^*2*^ = .25). Several parametric predictors exhibited associations. Increased age negatively predicted harsher judgments (*b* = -0.46, *SE* = 0.11, *t*(253) = -4.21, *p* < .001), and females (compared to males) provided more severe moral judgments (*b* = -7.67, *SE* = 3.42, *t*(253) = -2.24, *p* = .026). There was evidence for a marginal association when testing social media identity (*b* = -2.90, *SE* = 1.68, *t*(253) = -1.73, *p* = .085). Still, we could not reject the null hypothesis for social group (*b* = 3.60, *SE* = 3.43, *t*(253) = 1.05, *p* = .294).

Concerning the non-linear predictors, FoMO was associated with moral judgments (*edf* = 8.28, *F* = 3.75, *p* < .001) as was agreeableness (*edf* = 6.43, *F* = 3.23, *p* = .003). At lower values of FoMO (around 1.0 to 2.0), the effect on judgment appears relatively stable, with minor fluctuations around zero. However, as FoMO increases (above 2.0), a more pronounced increase in the judgment score (decreased severity) is observed, followed by a dip around values between 3.5 and 4.0. Beyond 4.0, the relationship rises sharply. If forced to be linear, the relationship shows a decrease in severity (increase in score) as FoMO rises.

*Personal recall*. The model explained 27.2% of the deviance (variance: *adjusted R*^*2*^ = .25). Among the parametric predictors, FoMO predicted personal recall (*b* = 0.36, *SE* = 0.08, *t*(265) = 4.55, *p* < .001), as did extraversion (*b* = 0.28, *SE* = 0.09, *t*(265) = 3.26, *p* = .001), religious fundamentalism recall (*b* = 0.09, *SE* = 0.03, *t*(265) = 3.25, *p* = .001) and need for cognition (*b* = 0.30, *SE* = 0.09, *t*(265) = 3.42, *p* < .001); increases in each had a greater likelihood of committing similar past moral violations. Conscientiousness was negatively associated with personal recall (*b* = -0.30, *SE* = 0.08, *t*(265) = -3.51, *p* < .001). Social group and personal recall were unrelated in this model (*b* = 0.08, *SE* = 0.12, *t*(265) = 0.66, *p* = .51). Focusing on the non-linear relationships, agreeableness (*edf* = 2.31, *F* = 5.24, *p* = .002) related to personal recall.

*Social recall*. The model explained 24% of the deviance (variance: *adjusted R*^*2*^ = .19). Several parametric predictors significantly affected social recall. Need for cognition and social recall were positively associated (*b* = 0.56, *SE* = 0.13, *t*(255) = 4.33, *p* < .001). Additionally, biracial individuals (*b* = 1.00, *SE* = 0.50, *t*(255) = 1.99, *p* = .047) exhibited greater social recall compared to White individuals and identifying as American Indian approached significance (*b* = 1.98, *SE* = 1.04, *t*(255) = 1.91, *p* = .057). Yet, we could not reject the null hypothesis when examining the relationship between social group and social recall (*b* = 0.05, *SE* = 0.19, *t*(255) = 0.29, *p* = .775).

Regarding non-linear predictors, FoMO showed an association (*edf* = 2.94, *F* = 5.74, *p* < .001), as did agreeableness (*edf* = 6.59, *F* = 3.22, *p* = .002) and religious fundamentalism (*edf* = 4.22, *F* = 2.50, *p* = .028).There is a steady, but not completely linear, increase in social recall as FoMO increases.

*Personal prediction*. The model explained 40% of the deviance (variance: *adjusted R*^*2*^ = .33). Notably, conscientiousness exhibited a negative association (*b* = -0.19, *SE* = 0.08, *t*(245) = -2.52, *p* = .013), while the need for cognition demonstrated a positive association (*b* = 0.26, *SE* = 0.08, *t*(245) = 3.27, *p* = .001). Furthermore, social group displayed an association (*b* = 0.21, *SE* = 0.11, *t*(245) = 1.97, *p* = .05).

For the non-linear predictors, FoMO (*edf* = 2.40, *F* = 4.44, *p* = .005), age (*edf* = 5.63, *F* = 3.28, *p* = .003), agreeableness (*edf* = 6.39, *F* = 3.28, *p* = .002), extraversion (*edf* = 4.42, *F* = 3.03, *p* = .01), and religious fundamentalism (*edf* = 4.75, *F* = 2.49, *p* = .023) each contributed unique variance to personal prediction. Personal prediction scores initially rise slightly with increasing FoMO but then level off at moderate levels with uncertainty at the higher end.

*Social prediction*. The model explained 20.2% of the deviance (variance: *adjusted R*^*2*^ = .16). The model yielded positive associations of FoMO (*b* = 0.43, *SE* = 0.12, *t*(258) = 3.70, *p* < .001) and need for cognition (*b* = 0.46, *SE* = 0.13, *t*(258) = 3.56, *p* < .001) with social prediction; social group failed to contribute unique variance (*b* = 0.03, *SE* = 0.18, *t*(258) = 0.19, *p* = .852). Political identity had a marginally significant negative association with social prediction (*b* = -0.09, *SE* = 0.05, *t*(258) = 1.86, *p* = .064). Additionally, agreeableness (*edf* = 6.13, *F* = 3.53, *p* = .001) and religious fundamentalism (*edf* = 4.62, *F* = 2.71, *p* = .015) each exhibited non-linear associations with social prediction.

#### Machine learning

*Moral judgment*. Among the models, SVR performed the best with an *R*^2^ value of .61 and an MAE of 15.10 for predicting moral judgment. All the models had an *R*^2^ higher than .56 and MAE no higher than 16.06. The features that carried the most weight were personal prediction (*M =* .41, *SD* = .18) and moral awareness (*M =* .29, *SD* = .09). Everything else was below .10.

**Fig 2:**
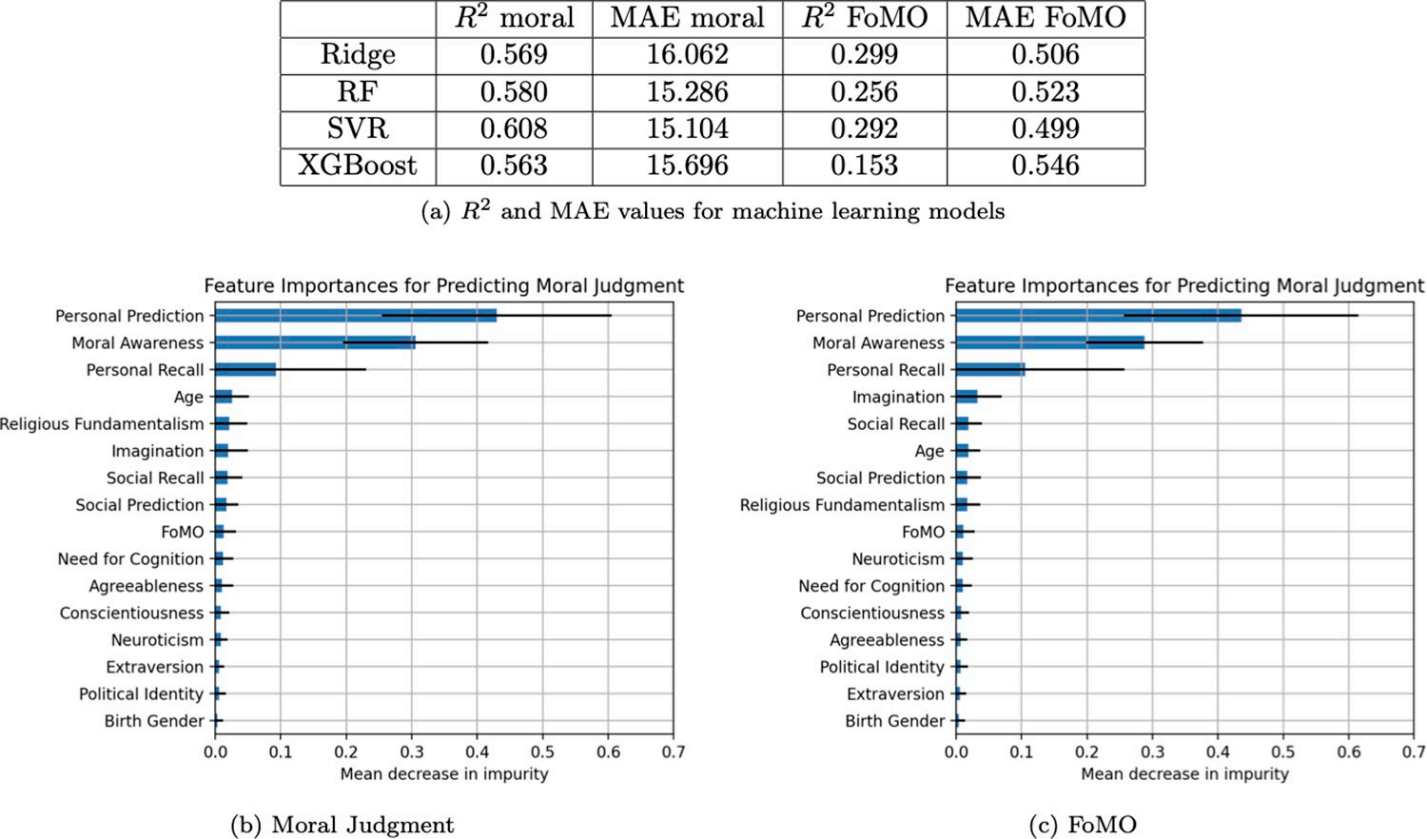
Study 1 machine learning performance. (A) R2 and MAE values for machine learning models. (B) Feature importances for predicting moral judgment. (C) Feature importances for predicting FoMO.

*FoMO*. Among the models, Ridge performed the best with respect to *R*^2^, producing a value of .30, while SVR produced the best MAE for predicting FoMO, with a value of 0.50. All the models had an *R*^2^ higher than .15 and MAE no higher than 0.55. The feature that carried the most weight was age (*M =* .36, *SD* = .11). Everything else was at or below .11. See Fig 2 below for full results.

### Discussion

Study 1 results support the hypothesized relationship between FoMO and moral judgments when using GAM and treating FoMO as a continuous variable. However, we could not reject the null hypothesis when examining whether FoMO was associated with moral awareness with RCM and GAM approaches. Likewise, we could not reject the null hypothesis when examining whether social setting was associated with moral judgments. These findings suggest a possible FoMO-moral judgment relationship exists for first-person moral violations where the participant is the perpetrator. When using a GAM approach, we found that those with higher FoMO reported higher levels of personal recall and prediction, as well as social recall and social prediction.

Beyond providing initial evidence of relationships between FoMO and moral outcomes of interest, we were able to predict moral judgment meaningfully well using machine learning approaches even though FoMO was not a very important feature compared to the top three features, which combined carried ~80% predictive importance. Approaching the relationship from the other direction, we could not predict trait FoMO with the same degree of success. However, moral judgment was the third most important feature.

## Study 2

In Study 2, we aimed to replicate and extend the findings of Study 1 to third-person moral judgments. Specifically, we again tested the relationship between FoMO and moral judgments but changed the agent’s identity in relation to the participant (stranger, friend, or someone disliked). Thus, we hypothesized that FoMO would be associated with lower moral awareness and less severe moral judgment. Additionally, we hypothesized that moral violations with a disliked agent would be associated with greater moral awareness and more severe moral judgments than strangers or liked agents. Likewise, we hypothesized that moral violations with a liked agent would be associated with less moral awareness and less severe moral judgments than strangers or disliked agents. Lastly, we hypothesized that FoMO would be associated with greater personal and social recall, as well as personal and social prediction.

### Methods

#### Participants

We recruited an approximately nationally representative panel of participants (N = 821) through Qualtrics (see Table 1). All participants gave informed consent electronically online. Institutional Review Board (IRB) approval was obtained from Duke University.

#### Procedures and materials

**Fig 3:**
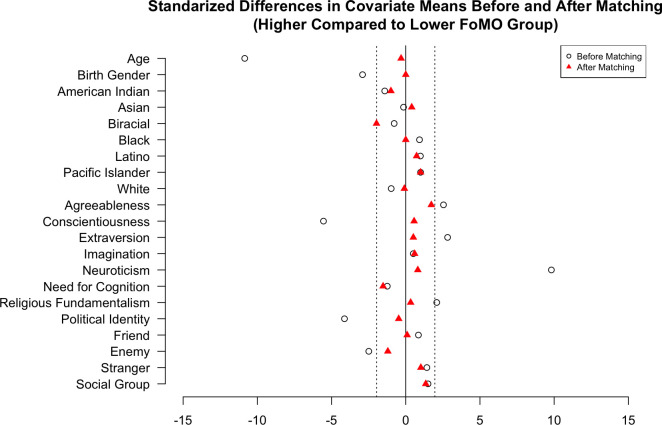
Study 2 covariate Love plot. Fig 3 shows the standardized differences in covariate means before (white circle) and after (red triangle) matching.

We used all the measures outlined in Study 1 without the social media identity measure and the addition of a new manipulation. Participants were randomly assigned to one of three agent conditions. The “like” condition asked the participant to enter the first name of someone they knew personally and liked very much as a friend. The “dislike” condition asked the participant to enter the first name of someone they knew personally, disliked very much, and was not a friend. The “stranger” condition asked them to enter the name of “Pat, a stranger.” Participants were then randomly assigned again to one of the two social conditions from Study 1. For our RCM treatment, the median FoMO value was 1.70. Before matching, the unbalanced covariates between groups were age, birth gender, agreeableness, conscientiousness, extraversion, neuroticism, religious fundamentalism, political identity, and enemy. After matching with a caliper of .2 (standard deviation of the estimated propensity score), we could no longer observe any evidence of imbalance for these variables (see Fig 3). This matching decreased the sample size from 821 to 496 participants.

For the GAM, we wanted to remain consistent with our previous analyses and included only participants with moral awareness and judgment scores greater than 2.5 and less than 50, respectively. Thus, our Fisher exact tests used all 496 matched participants, while our GAM models used data from 805 participants.

#### Analysis

All statistical analyses followed the same steps as Study 1 to find further support for our previous results. The ML approaches were also the same as in Study 1.

### Results

#### Rubin causal model

Our Fisher tests found evidence that those with higher levels of FoMO gave less severe judgments of moral violations and predicted engaging in more moral violations in the future compared to their lower FoMO peers. Also of note is that personal recall approached statistical significance, suggesting that those with higher FoMO have engaged in more moral violations in the past than those with lower FoMO. See Table 4 for full results.

**Table 4:** Study 2 Fisher test results.

	Higher FoMO	Lower FoMO	Estimated Average Causal Effect	Fisher p-value	95% Fisherian Intervals
Moral Awareness	5.41 (0.91)	5.42 (0.98)	-0.01	.96	[-0.19, 0.18]
Moral Judgment	-54.39 (35.64)	-61.87 (27.30)	7.48	.0091	[1.10, 13.88]
Personal Recall	2.24 (1.26)	2.05 (1.07)	0.19	.0633	[-0.04, 0.43]
Social Recall	3.43 (1.59)	3.31 (1.59)	0.12	.4078	[-0.20, 0.43]
Personal Prediction	2.13 (1.20)	1.88 (0.91)	0.26	.0068	[0.05, 0.47]
Social Prediction	3.36 (1.52)	3.25 (1.50)	0.11	.4091	[-0.19, 0.41]

Note. Means were presented for all outcome variables for both treatment arms, with standard deviations in parentheses. Fisher p-values are based on 100,000 permutations as are the Fisherian Intervals.

#### Generalized additive model

*Moral awareness*. The model explained 19% of the deviance (variance: *adjusted R*^*2*^ = .17). Among the parametric predictors, individuals identifying as Black had lower awareness scores compared to White participants (*b* = -0.26, *SE* = 0.09, *t*(785) = -2.77, *p* = .006). Likewise, being Latino associated with lower moral awareness (*b* = -0.24, *SE* = 0.08, *t*(785) = -3.12, *p* = .002) compared to White individuals. Although the agent being a friend was a significant negative predictor (*b* = -0.17, *SE* = 0.07, *t*(785) = -2.47, *p* = .014), we could not reject the null hypothesis when the agent was disliked (*b* = 0.00, *SE* = 0.07, *t*(785) = 0.00, *p* = .997). Extraversion approached significance (*b* = -0.06, *SE* = 0.03, *t*(785) = -1.69, *p* = .092), as did social group (*b* = 0.09, *SE* = 0.06, *t*(785) = 1.64, *p* = .102).

When analyzing the non-linear predictors, we again could not reject the null hypothesis for a relationship between FoMO and moral awareness (*edf* = 2.66, *F* = 1.64, *p* = .157), while conscientiousness displayed a potentially positive effect (*edf* = 4.86, *F* = 2.05, *p* = .071). Only need for cognition (*edf* = 3.71, *F* = 16.87, *p* < .001) and religious fundamentalism (*edf* = 3.04, *F* = 11.07, *p* < .001) predicted moral awareness among the non-linear predictors.

*Moral judgment*. The model explained 12.2% of the deviance (variance: *adjusted R*^*2*^ = .11). FoMO positively predicted less severity of moral judgment (*b* = 3.66, *SE* = 1.21, *t*(794) = 3.01, *p* = .003). Similarly, the agent as a friend compared to a stranger led to less severe moral judgments (*b* = 5.77, *SE* = 2.34, *t*(794) = 2.47, *p* = .014); we could not reject the null hypothesis when the agent was disliked (*b* = -0.58, *SE* = 2.51, *t*(794) = -0.23, *p* = .819). Social group exhibited a possible influence (*b* = 3.82, *SE* = 2.00, *t*(794) = 1.91, *p* = .057). Regarding the non-linear predictors, only religious fundamentalism exhibited an effect on judgment (*edf* = 7.03, *F* = 10.23, *p* < .001).

*Personal recall*. The model explained 7.89% of the deviance (variance: *adjusted R*^*2*^ = .07). FoMO was positively associated with personal recall (*b* = 0.16, *SE* = 0.05, *t*(795) = 3.26, *p* = .001), along with agreeableness (*b* = 0.13, *SE* = 0.06, t(795) = 2.32, *p* = .021). We found evidence of a possible relationship of social group (*b* = 0.15, *SE* = 0.08, *t*(795) = 1.85, *p* = .064), yet we could not reject the null hypothesis for when the agents were friends (*b* = 0.10, *SE* = 0.09, *t*(795) = 1.05, *p* = .293), nor disliked (*b* = -0.08, *SE* = 0.10, *t*(795) = -0.83, *p* = .407) compared to strangers. Examining the non-linear predictors, need for cognition (*edf* = 2.54, *F* = 2.84, *p* = .035) and religious fundamentalism (*edf* = 2.39, *F* = 10.43, *p* < .001) associated with personal recall.

*Social recall*. The model explained 7.5% of the deviance (variance: *adjusted R*^*2*^ = .06). FoMO displayed a positive association with social recall (*b* = 0.18, *SE* = 0.07, t(793) = 2.61, *p* = .009). Additionally, individuals identifying as biracial exhibited higher social recall than White individuals (*b* = 0.73, *SE* = 0.32, *t*(793) = 2.30, *p* = .022). Agreeableness (*b* = 0.17, *SE* = 0.08, *t*(793) = 2.03, *p* = .043) and need for cognition (*b* = 0.25, *SE* = 0.08, *t*(793) = 3.11, *p* = .002) also positively related to social recall. In contrast, religious fundamentalism was associated with lower social recall (*b* = -0.10, *SE* = 0.03, *t*(793) = -3.79, *p* < .001). When agents were disliked, participants had higher social recall (*b* = 0.29, *SE* = 0.14, *t*(793) = 2.10, *p* = .036), but we could not reject the null hypothesis for friends (*b* = 0.04, *SE* = 0.13, *t*(793) = 0.34, *p* = .736). Social group approached significance (*b* = 0.19, *SE* = 0.11, *t*(793) = 1.74, *p* = .082). Additionally, the analysis identified a non-linear effect of extraversion (*edf* = 4.01, *F* = 2.84, *p* = .016).

*Personal prediction*. The model explained 11.9% of the deviance (variance: *adjusted R*^*2*^ = .11). FoMO predicted higher personal prediction (*b* = 0.19, *SE* = 0.04, *t*(794) = 4.49, *p* < .001), as did being in the highly social group (*b* = 0.15, *SE* = 0.07, *t*(794) = 2.20, *p* = .028). However, we could not reject the null hypothesis for when the agents were friends (*b* = 0.08, *SE* = 0.08, *t*(794) = 1.05, *p* = .296) or disliked (*b* = -0.07, *SE* = 0.09, *t*(794) = -0.84, *p* = .402) compared to strangers. Need for cognition (*edf* = 4.00, *F* = 6.68, *p* < .001) and religious fundamentalism (*edf* = 2.97, *F* = 9.40, *p* < .001) did exhibit non-linear effects.

*Social prediction*. The model explained 6.51% of the deviance (variance: *adjusted R*^*2*^ = .05). FoMO positively associated with social prediction (*b* = 0.18, *SE* = 0.07, *t*(793) = 2.78, *p* = .006) as did being biracial (*b* = 0.70, *SE* = 0.31, *t*(793) = 2.27, *p* = .024) compared to White. Need for cognition also positively associated with social prediction (*b* = 0.20, *SE* = 0.08, *t*(793) = 2.59, *p* = .010), while religious fundamentalism negatively associated with such predictions (*b* = -0.08, *SE* = 0.03, *t*(793) = -3.06, *p* = .002. The agent being disliked, rather than a stranger, influenced social prediction (*b* = 0.28, *SE* = 0.14, *t*(793) = 2.10, *p* = .036). However, we could not reject the null hypotheses when the agent was a friend (*b* = -0.01, *SE* = 0.13, *t*(793) = -0.07, *p* = .948) or for being in the highly social group (*b* = 0.16, *SE* = 0.11, *t*(793) = 1.47, *p* = .143). For those that identified as American Indian, compared to White, the relationship approached significance (*b* = -1/78, *SE* = 1.08, *t*(793) = -1.65, *p* = .099), Additionally, extraversion displayed a non-linear effect (*edf* = 4.13, *F* = 2.49, *p* = .03).

#### Machine learning

*Moral judgment*. Among the models, RF performed the best with an *R*^2^ value of .63 and an MAE of 13.80 for predicting moral judgment. All the models had an *R*^2^ higher than .57 and an MAE no higher than 15.53. The features that carried the most weight were personal prediction (*M* = .47, *SD* = .11) and moral awareness (*M* = .33, *SD* = .09). Everything else was below .10.

*FoMO*. Among the models, Ridge performed the best with respect to *R*^2^, producing a value of .28, while SVR produced the best MAE for predicting FoMO, with a value of 0.53.

**Fig 4:**
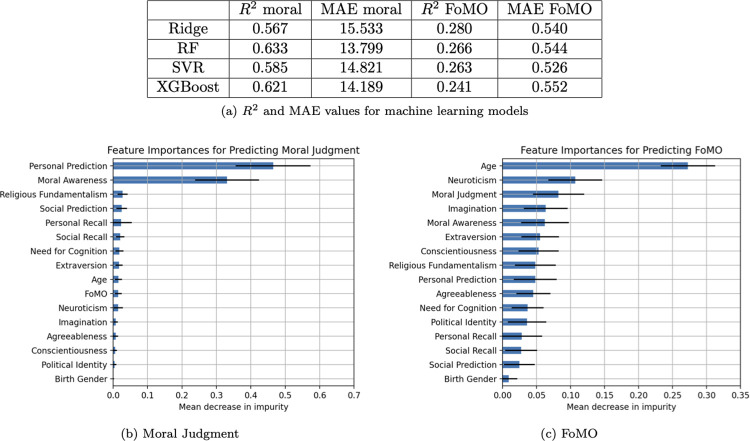
Study 2 machine learning performance. (A) R2 and MAE values for machine learning models. (B) Feature importances for predicting moral judgment. (C) Feature importances for predicting FoMO.

All the models had an *R*^2^ higher than .24 and an MAE no higher than 0.55. The feature that carried the most weight was age (*M* = .27, *SD* = .04). Everything else was at or below .11. See Fig 4 below for full results.

### Discussion

Replicating and extending the results of Study 1, we found additional support for the hypothesized relationships that higher FoMO associates with less severe judgments for moral violations, greater recall of past transgressions (their own and others), and stronger expectations for future moral violations (their own and others). When we used GAM and FoMO as a continuous variable, those with higher levels of FoMO reported greater moral awareness. The agent being a friend (compared to a stranger) led to less severe moral judgments supporting one of our hypotheses about agent identity. Similarly, highly social settings closely approached significance for decreasing the severity of moral judgments, not confirming our hypothesis but suggesting that such a relationship might exist.

Again, we could predict the average moral judgment across violations for an individual with even better performance. Personal prediction and moral awareness accounted for over 70% of the combined predictive power. Our ability to predict FoMO increased as well compared to Study 1. Age, personal prediction, and moral judgment were the top three features in determining which FoMO score to predict. In sum, Study 2 confirms findings from Study 1 while effectively extending those results to include third-person moral violations involving a stranger, friend, or disliked agent.

## Study 3

For Study 3, we aimed to replicate the findings of Studies 1 and 2 while extending them via a FoMO manipulation. We attempted to manipulate FoMO via a recall methodology to examine moral judgments after recalling a FoMO experience (FoMO condition) or not (no FoMO condition). We expected the high FoMO condition to report less severe moral judgments than the no FoMO condition. All other hypothesized relationships remained the same.

### Method

#### Participants and design

We recruited an approximately nationally representative panel of participants (N = 604) through Qualtrics (see Table 1). All participants gave informed consent electronically online. Institutional Review Board (IRB) approval was obtained from Duke University.

#### Procedures and materials

**Fig 5:**
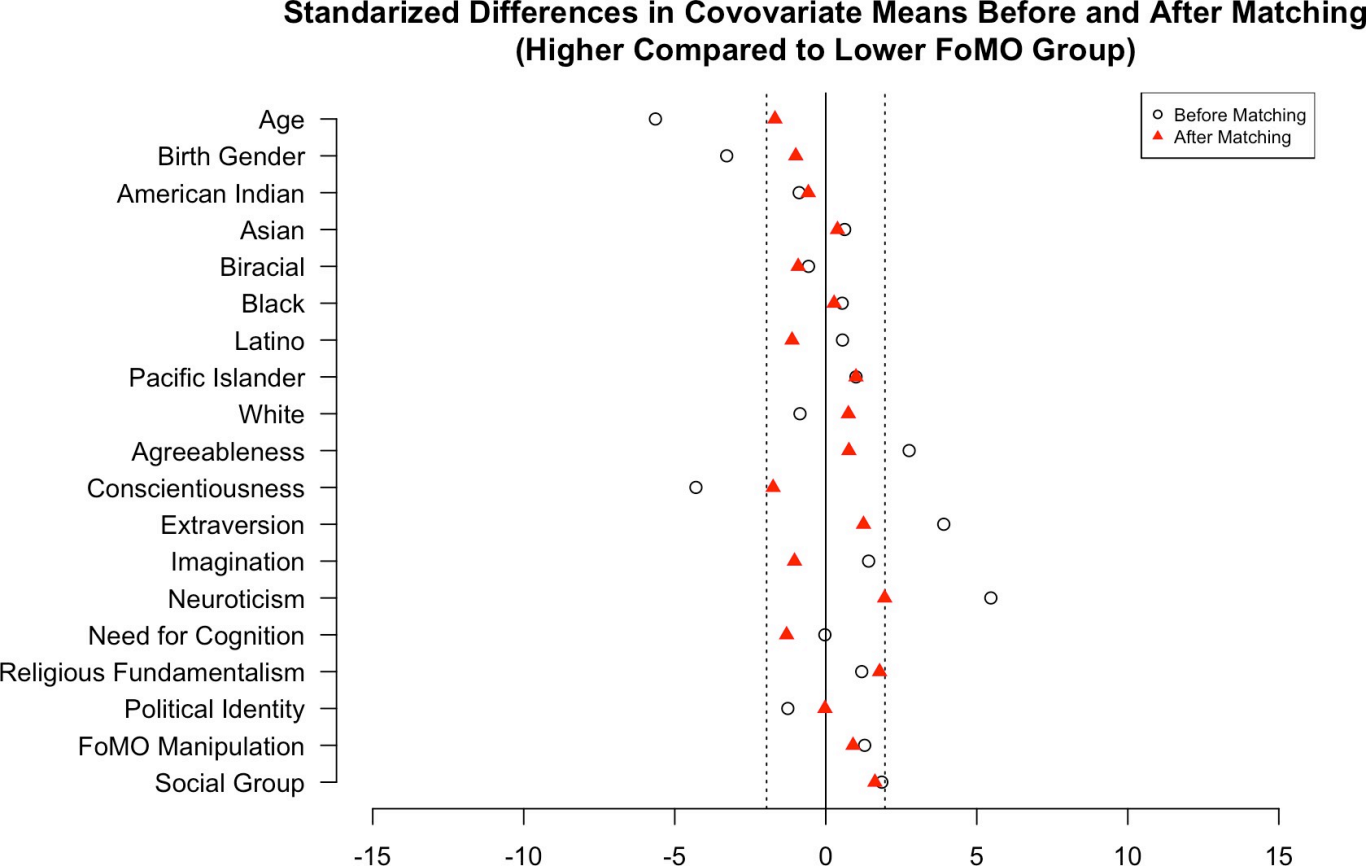
Study 3 covariate Love plot. Fig 5 shows the standardized differences in covariate means before (white circle) and after (red triangle) matching.

Study 3 used all the measures from Study 2 without the agent conditions (i.e., first-person violations like Study 1) and with the addition of a new manipulation. Participants were randomly assigned to one of two recall conditions. The “FoMO” condition asked participants to recall and describe in a few sentences a time they missed out on a fun activity that friends or family were engaging in, why they missed it, and how they felt about missing it. The “no FoMO” condition asked the participant to recall and describe in a few sentences a time they engaged in a fun activity with family or friends, why the activity took place, and how they felt about engaging in the activity. Participants were then randomly assigned again to one of the two social conditions from Study 1. For this study, we used the FoMO score taken after the recall conditions for the median, which was 1.78. Before matching, the unbalanced covariates between groups were age, birth gender, agreeableness, conscientiousness, extraversion, and neuroticism. After matching with a caliper of .4 (standard deviation of the estimated propensity scores), we could not observe any evidence of imbalance for these variables (see Fig 5). This matching decreased the sample size from 604 to 490 participants.

Again, for our GAM, we wanted to remain consistent with our previous analyses and included only participants with moral awareness and judgment scores greater than 2.5 and less than 50, respectively. Thus, our Fisher exact tests used all 490 matched participants, while our GAM models used 591 participants.

### Analysis

All analyses followed the same steps as Study 2.

### Results

#### FoMO recall manipulation

When looking at those who recalled a time they did not miss out on an opportunity, we could not reject the null hypothesis of there being no change of FoMO before and after the recall (Mean Difference = -0.02, *SE* = 0.02, *t*[291] = -0.96, *p* = .34, 95% *CI* = [-0.06, 0.02]). However, when looking at those who did recall a time they missed out on an opportunity, we found an increase in FoMO after the recall (Mean Difference = 0.07, *SE* = 0.03, *t*[311] = 2.94, *p* = .004, 95% *CI* = [0.02, 0.12]).

#### Rubin causal model

Our Fisher tests comparing our FoMO treatment arms indicate group differences in moral judgment, personal recall, social recall, personal prediction, and social prediction. Specifically, the higher FoMO group reported less severe judgments, a greater likelihood of having done a similar violation in the past while also being more likely to commit a similar violation in the future, as well as a greater likelihood of knowing someone personally who has done a similar violation in the past and knowing someone likely to commit similar violations in the future. A relationship for moral awareness approached statistical significance with a possible negative effect for higher FoMO. See Table 5 for full results.

**Table 5:** Study 3 Fisher test results.

	Higher FoMO	Lower FoMO	Estimated Average Causal Effect	Fisher p-value	95% Fisherian Intervals
Moral Awareness	5.33 (0.93)	5.48 (0.99)	-0.16	.0707	[-0.35, 0.03]
Moral Judgment	-48.01 (38.90)	-60.45 (30.85)	12.43	< .0001	[5.29, 19.55]
Personal Recall	2.49 (1.29)	2.07 (1.07)	0.43	< .0001	[0.19, 0.66]
Social Recall	3.62 (1.46)	3.29 (1.59)	0.33	.0174	[0.02, 0.63]
Personal Prediction	2.43 (1.22)	1.96 (0.97)	0.47	< .0001	[0.25, 0.69]
Social Prediction	3.63 (1.39)	3.29 (1.56)	0.34	.0113	[0.04, 0.63]

Note. Means were presented for all outcome variables for both treatment arms, with standard deviations in parentheses. Fisher p-values are based on 100,000 permutations as are the Fisherian Intervals.

#### Generalized additive model

*Moral awareness*. The model explained 27.9% of the deviance (variance: *adjusted R*^*2*^ = .25). Among the parametric predictors, participants’ age (*b* = 0.01, *SE* = 0.00, *t*(569) = 4.18, *p* < .001), being female (*b* = 0.28, *SE* = 0.07, *t*(569) = 3.81, *p* < .001), agreeableness (*b* = 0.14, *SE* = 0.05, *t*(569) = 2.71, *p* = .007), and having a more conservative political identity (*b* = 0.07, *SE* = 0.02, *t*(569) = 3.27, *p* = .001) exhibited significant positive relationships with awareness. However, being Black (compared to White) was negatively associated with moral awareness (*b* = -0.32, *SE* = 0.11, *t*(569) = -2.88, *p* = .036) as was being in the FoMO recall condition (*b* = -0.14, *SE* = 0.07, *t*(569) = -2.10, *p* = .036). We could not reject the null hypotheses for FoMO (*b* = -0.07, *SE* = 0.05, *t*(569) = -1.62, *p* = .108) or social group (*b* = 0.3, *SE* = 0.07, *t*(569) = 0.49, *p* = .626). As non-linear predictors, extraversion demonstrated statistical significance (*edf* = 3.90, *F* = 2.42, *p* = .033), as did need for cognition (*edf* = 7.74, *F* = 4.07, *p* < .001), and religious fundamentalism (*edf* = 2.26, *F* = 3.33, *p* = .017).

*Moral judgment*. The model explained 28.6% of the deviance (variance: *adjusted R*^*2*^ = .26). Among the parametric predictors, several variables demonstrated relationships. Being female (as opposed to male; *b* = -13.65, *SE* = 2.41, *t*(569) = -5.66, *p* < .001), having increased conscientiousness (*b* = -3.87, *SE* = 1.61, *t*(569) = -2.40, *p* = .017), and being more conservative (political identity; *b* = -2.91, *SE* = 0.68, *t*(569) = -4.29, *p* < .001) associated with more severe moral judgments. Additionally, FoMO manipulation’s recalling of a missed opportunity was associated with less severe moral judgments (*b* = 6.20, *SE* = 2.27, *t*(569) = 2.74, *p* = .006). Being American Indian, compared to White, approached significance (*b* = 26.21, *SE* = 13.80, *t*(569) = 1.90, *p* = .058). In terms of non-linear predictors, FoMO (*edf* = 2.11, *F* = 11.50, *p* < .001), agreeableness (*edf* = 4.59, *F* = 4.35, *p* < .001), extraversion (*edf* = 2.71, *F* = 3.33, *p* = .013), and need for cognition (*edf* = 6.47, *F* = 2.72, *p* = .006) all had evidence in support of non-linear relationships with moral judgments. FoMO’s relationship is relatively close to being linear, with a decrease in judgment severity as FoMO increases.

*Personal recall*. The model explained 28.5% of the deviance (variance: *adjusted R*^*2*^ = .26). The results suggest that FoMO was positively associated with personal recall (*b* = 0.27, *SE* = 0.06, *t*(569) = 4.83, *p* < .001) as were extraversion (*b* = 0.23, *SE* = 0.05, *t*(569) = 4.97, *p* < .001), neuroticism (*b* = 0.15, *SE* = 0.05 *t*(569) = 2.74, *p* = .006), and being American Indian (*b* = 1.29, *SE* = 0.48, *t*(569) = 2.66, *p* = .008). Females reported lower personal recall than males (*b* = -0.23, *SE* = 0.09, *t*(569) = -2.66, *p* = .008). Higher conscientiousness (*b* = -0.20, *SE* = 0.06, *t*(569) = -3.20, *p* = .001) and religious fundamentalism (*b* = -0.05, *SE* = 0.02, *t*(569) = -2.42, *p* = .016) also predicted lower personal recall. We could not reject the null hypotheses for social group (*b* = 0.09, *SE* = 0.08, *t*(569) = 1.09, *p* = .275) or FoMO manipulation (*b* = 0.04, *SE* = 0.08, *t*(569) = 0.45, *p* = .656). In terms of non-linear predictors, age (*edf* = 8.21, *F* = 2.46, *p* = .014) and agreeableness (*edf* = 4.43, *F* = 5.30, *p* < .001) both demonstrated non-linear relationships with personal recall.

*Social recall*. The model explained 22.2% of the deviance (variance: *adjusted R*^*2*^ = .19). Higher neuroticism (*b* = 0.34, *SE* = 0.08, *t*(570) = 4.42, *p* < .001), more conservative political identity (*b* = 0.09, *SE* = 0.04, *t*(570) = 2.35, *p* = .019), and need for cognition (*b* = 0.25, *SE* = 0.10, *t*(570) = 2.42, *p* = .016) each predicted greater social recall. Religious fundamentalism (*b* = -0.11, *SE* = 0.03, *t*(570) = -3.44, *p* < .001), however, negatively predicted social recall. Conscientiousness (*b* = -0.17, *SE* = 0.09, *t*(570) = -1.89, *p* = .059) and imagination (*b* = 0.18, *SE* = 0.10, *t*(570) = 1.76, *p* = .079) approached statistical significance. Identifying as Black (*b* = -0.34, *SE* = 0.19, *t*(570) = -1.79, *p* = .074), being in the highly social group (*b* = 0.04, *SE* = 0.12, *t*(570) = 0.37, *p* = .709), or recalling a missed opportunity (FoMO manipulation; *b* = -0.09, *SE* = 0.12, *t*(570) = -0.80, *p* = .423) were not significant predictors.

Concerning the non-linear models, FoMO (*edf* = 2.15, *F* = 5.33, *p* = .003), age (*edf* = 2.24, *F* = 2.34, *p* = .068), agreeableness (*edf* = 2.46, *F* = 4.55, *p* = .004), and extraversion (*edf* = 4.77, *F* = 3.64, *p* = .002) all demonstrated non-linear relationships with social recall. For FoMO, social recall increases with moderate levels of FoMO with the relationship leveling off at higher levels.

*Personal prediction*. The model explained 31.8% of the deviance (variance: *adjusted R*^*2*^ = .29). Specifically, FoMO (*b* = 0.28, *SE* = 0.05, *t*(568) = 5.66, *p* < .001) and identifying as an American Indian (as opposed to White; *b* = 1.61, *SE* = 0.43, *t*(568) = 3.70, *p* < .001) each resulted in higher personal predictions of future morally wrong behavior. However, being a female (compared to a male; *b* = -0.29, *SE* = 0.08, *t*(568) = -3.82, *p* < .001), conscientiousness (*b* = -0.19, *SE* = 0.06, *t*(568) = -3.44, *p* < .001), and religious fundamentalism (*b* = -0.04, *SE* = 0.02, *t*(568) = -2.18, *p* = .03) each associated with fewer predictions of future moral violations. Neuroticism approached significance (*b* = 0.09, *SE* = 0.05, *t*(568) = 1.90, *p* = .058). Still, we could not reject the null hypotheses for relationships with social group (*b* = 0.10, *SE* = 0.07, *t*(568) = 1.35, *p* = .176) or FoMO manipulation (*b* = 0.10, *SE* = 0.07, *t*(568) = 1.44, *p* = .15).

In addition to parametric predictors, non-linear predictors were also found. Age (*edf* = 7.38, *F* = 2.29, *p* = .024), agreeableness (*edf* = 4.54, *F* = 4.70, *p* < .001), and extraversion (*edf* = 3.06, *F* = 4.72, *p* = .001) all exhibited non-linear relationships.

*Social prediction*. The model explained 17.8% of the deviance (variance: *adjusted R*^*2*^ = .16). Neuroticism (*b* = 0.40, *SE* = 0.07, *t*(577) = 5.87, *p* < .001) and the need for cognition (*b* = 0.29, *SE* = 0.09, *t*(577) = 3.21, *p* = .001) each predicted more expected moral violations from people they know. However, religious fundamentalism negatively predicted social recall scores (*b* = -0.08, *SE* = 0.03, *t*(577) = -2.83, *p* = .005). We could not reject the null hypotheses for relationships with social group (*b* = 0.00, *SE* = 0.11, *t*(577) = 0.01, *p* = .989) or FoMO manipulation (*b* = -0.06, *SE* = 0.11, *t*(577) = -0.563, *p* = .573).

Regarding non-linear prediction, FoMO (*edf* = 2.26, *F* = 5.70, *p* = .001), agreeableness (*edf* = 2.79, *F* = 3.92, *p* = .008), and extraversion (*edf* = 4.40, *F* = 3.69, *p* = .002) all demonstrated nonlinear social prediction relationships. Regarding FoMO, social prediction increases at moderate levels of FoMO, but levels off at higher FoMO scores.

#### Machine learning

*Moral judgment*. Among the models, RF performed the best (*R*^2^ = .60; MAE = 14.23) when predicting moral judgment. All models’ *R*^2^ exceeded .56 with MAEs no higher than 15.89. The features personal prediction (*M* = .51, *SD* = .11) and moral awareness (*M* = .31, *SD* = .10) carried the most weight. The remaining features were below .03.

**Fig 6:**
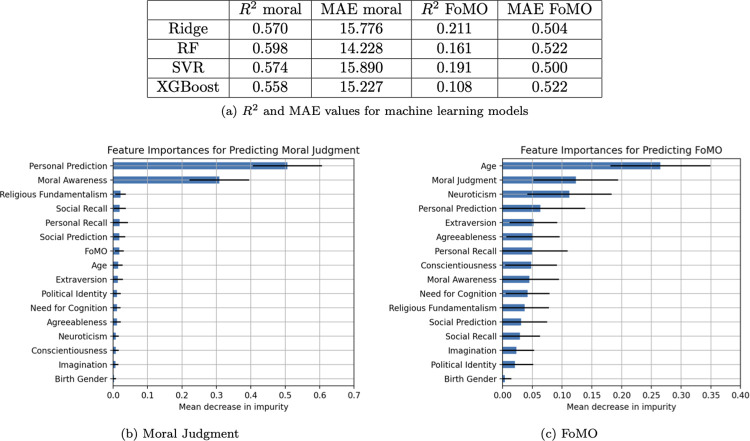
Study 3 machine learning performance. (A) R2 and MAE values for machine learning models. (B) Feature importances for predicting moral judgment. (C) Feature importances for predicting FoMO.

*FoMO*. Among the models, Ridge performed the best among the tested models (*R*^2^ = .21), while with respect to MAE with a value of 0.50, SVR performed the best. All models had an *R*^2^ greater than .11 and an MAE less than 0.52. The feature that carried the most weight was age (*M* = .26, *SD* = .09); all remaining features remained less than .13. See Fig 6 below for full results.

### Discussion

Consistent with both previous studies, higher FoMO individuals reported less severe moral violation judgments when we used RCM and GAM. Further corroborating Study 1 and 2’s results, we also found that higher FoMO levels predicted more personal and social recall of past moral violations and more personal and social predictions regarding future moral transgressions. Moreover, recalling a missed experience decreased moral awareness and reduced the severity of moral judgments compared to recalling engagement in a rewarding experience. These results also confirm important baseline differences in those that have varying FoMO levels and that recall procedures may effectively manipulate FoMO levels among individuals, even as measured with trait instead of state measures.

## General discussion

Using causal inference (RCM), traditional statistical analyses (GAM), and machine learning methods, we investigated whether FoMO had any relationships with an array of moral outcomes (e.g., awareness, judgment, and behaviors). We found convergent evidence across three studies that FoMO has relationships with moral judgments and behaviors. These findings were robust, remaining consistent whether the agents committing moral violations were the self (Study 1), a stranger, a friend, or someone known but disliked (Study 2). Additionally, findings remained robust whether participants recalled engaging in an enjoyable social activity (non-FoMO-inducing manipulation) or a time they missed such an opportunity (FoMO-inducing manipulation; Study 3). See Tables 6 and 7 below for summaries of findings.

**Table 6:** Summary of Fisher test results across all three studies.

Outcome	Study 1	Study 2	Study 3
**Awareness (1–7)**	-0.25 (.1354)	-0.01 (.9600)	-0.16 (.0707)
**Judgment (-100–100)**	8.06 (.1680)	**7.48 (.0091)**	**12.43 (< .0001)**
**Personal Recall (1–7)**	0.28 (.1515)	0.19 (.0633)	**0.43 (< .0001)**
**Social Recall (1–7)**	0.11 (.6868)	0.12 (.4078)	**0.33 (.0174)**
**Personal Prediction (1–7)**	0.27 (.1092)	**0.26 (.0068)**	**0.47 (< .0001)**
**Social Prediction (1–7)**	0.15 (.5724)	0.11 (.4091)	**0.34 (.0113)**

Note. Estimated average causal effect (Higher FoMO–Lower FoMO group) and Fisher p-value (in parentheses) for each study. Bold denotes p < .05. Scale is below outcome in parentheses for reference.

**Table 7:** Summary of GAM results for higher FoMO across all three studies.

	Study	Relationship	Direction	Meaning
**Moral Awareness (MA)**	1	No	-	-
	**2**	**Yes**	**Positive**	**Higher FoMO → greater MA**
	3	No	-	-
**Moral Judgment (MJ)**	**1**	**Yes**	**Positive**	**Higher FoMO → less severe MJ**
	**2**	**Yes**	**Positive**	**Higher FoMO → less severe MJ**
	**3**	**Yes**	**Positive**	**Higher FoMO → less severe MJ**
**Personal Recall (PR)**	**1**	**Yes**	**Positive**	**Higher FoMO → greater PR**
	**2**	**Yes**	**Positive**	**Higher FoMO → greater PR**
	3	No	-	-
**Social Recall (SR)**	**1**	**Yes**	**Positive**	**Higher FoMO → greater SR**
	**2**	**Yes**	**Positive**	**Higher FoMO → greater SR**
	**3**	**Yes**	**Positive**	**Higher FoMO → greater SR**
**Personal Prediction (PP)**	**1**	**Yes**	**Positive**	**Higher FoMO → greater PP**
	**2**	**Yes**	**Positive**	**Higher FoMO → greater PP**
	**3**	**Yes**	**Positive**	**Higher FoMO → greater PP**
**Social Prediction (SP)**	**1**	**Yes**	**Positive**	**Higher FoMO → greater SP**
	**2**	**Yes**	**Positive**	**Higher FoMO → greater SP**
	**3**	**Yes**	**Positive**	**Higher FoMO → greater SP**

### FoMO and moral awareness

Based upon relevant theory [11–13], we expected both FoMO and social setting to influence moral awareness, such that higher FoMO would lead to a decrease and highly social contexts each would lead to increased moral awareness. However, the results for those predictions varied somewhat depending on the analysis conducted. Results obtained via RCM found no difference between high and low FoMO groups across analyses, although non-significant mean differences trended in the expected direction. Yet the GAM from Study 2 suggested a possible positive relationship, and when FoMO was manipulated via recall, the FoMO condition displayed significantly higher awareness than the no FoMO condition. Although these differing results might simply be artifacts of the analysis choices made, they may also suggest the presence of potential moderators of the FoMO-moral awareness relationship, a potentially profitable avenue for future research [47].

Still, examining the descriptive statistics shows that moral awareness was relatively high for all participants regardless of trait FoMO level or social setting. Thus, considering COR and other social resource theories, all moral violations include the possibility of resource loss (or gain) regardless of FoMO levels or the social context. Additionally, we designed the moral violation vignettes to clearly and unambiguously be perceived as morally valenced. Selecting more subtle or ambiguous moral violations might be necessary to adequately assess the relationship between the focal variables of this work and moral awareness. Currently, the present results render support for such relationships equivocal, so future research in this area is required to better specify the influences on individuals’ moral awareness.

### FoMO and moral judgment

Based on past work arguing morality is inherently social [48, 49] and moral judgment is highly sensitive to the situation, context, and social consensus [18, 19, 25, 26], we hypothesized that FoMO and social setting would influence moral judgments by decreasing the severity. Supporting that prediction, we observed that higher FoMO predicted less severe judgments–judgments shifted closer to neutral. One possible explanation for this finding resides in resource-protective behavior. High FoMO individuals may be especially sensitive to social resource loss/gain and may adjust their notions of right and wrong to align with current social consensus to gain, avoid losing, or maintain current resources. For example, if one initially believes behavior is immoral but most of their social connections (e.g., friends, family) engage in that behavior, they may alter their position towards a less severe judgment. Marton-Alper and colleagues [50] found that even without the social group members’ physical presence, individuals practiced conformity to that group’s morals. This judgment shift, often referred to as moral conformity, has been found to occur when there is motivation to avoid social isolation via conformity or to be informationally correct [51–53].

In this context, those with FoMO may benefit from being less morally strict with their judgments, as higher moral strictness could limit their social options by setting a higher moral threshold, which is more difficult to achieve. Furthermore, FoMO could impact moral judgments through strategic self-presentation [54]. Individuals experiencing FoMO may be more inclined to engage in strategic self-presentation to enhance their social standing and avoid missing out on rewarding experiences. This could involve adjusting their moral judgments to align with the values and expectations of their social groups, thereby ingratiating themselves with others and increasing their chances of inclusion [55].

Additionally, individuals might use post-hoc reasoning to shield themselves from negative self-attributions while maintaining social relationships to preserve or gain valued social resources [27, 56]. They will do what they must (i.e., moral violations) to fit in with a group and maintain social resources, but ultimately, to not feel bad about themselves, they rationalize their behavior so they don’t have to think of themselves, the people they are with, or the things they are doing as bad–which would otherwise motivate them to stop.

Our findings also indicate that FoMO results in less severe judgments of moral violations regardless of interpersonal perspective (i.e., first-person or third-person judgments) or relationship status (i.e., stranger, friend, or a disliked other). As the moral domain is not a unilateral construct, individuals with high FoMO might experience conflicting moral values when facing social situations, such as the trade-off between overall fairness norms and loyalty to specific social targets [57, 58]. Consequently, FoMO may lead to a general decrease in some moral judgments and an increase in others, such as those related to defending and supporting one’s friends [24]. However, social context only approached a significant association with moral judgments in Study 2, such that being in the presence of best friends resulted in less severe moral judgments. Future work considering constructs such as self-other overlap or degree of closeness may help elucidate the mechanisms behind FoMO’s influence on moral judgments [59].

### FoMO and personal/social recall/prediction of moral violations

Higher FoMO individuals were more likely to recall their own and their social network’s past moral violations. Logically, this fits well with our other findings, as individuals judging immoral behavior less severely may also be more likely to engage in similar moral violations. This raises two competing causal sequences: 1) higher FoMO may reduce judgment severity to increase immoral behavior (i.e., the behavior does not seem that ‘bad’ so I can do it), or 2) higher FoMO may result in higher rates of immoral behavior and judgments may reflect post-hoc rationalizations to protect self-concepts or reputational resources. In more formal terms, either 1) judgment severity mediates the FoMO-immoral behavior relationship, or 2) immoral behavior may mediate the FoMO-post-hoc judgment relationship. Admittedly other options may exist, and future research will be needed for determining which of these causal sequences is more likely.

Consistent with the above logic, we additionally observed that higher FoMO levels predicted stronger personal prediction to engage in future moral violations (i.e., behavioral intent) and stronger beliefs that one’s social network is likely to engage in moral transgressions in the future. Thus, not only are higher FoMO individuals more likely to have engaged in their own and associate with people who engage in moral violations while judging those violations less severely, they also hold stronger intentions to commit moral violations in the future and expect the same from their social interaction partners. Such intentions might simply reflect high FoMO individuals’ strong drive to secure new social resources while avoiding the loss of current social resources [13]. To remain connected and involved so as not to miss social opportunities, high FoMO individuals may also be more likely to succumb to peer pressure. Given that high FoMO individuals exhibit greater recall of past violations, they may also anchor on those expectations when predicting the likelihood of future violations. High FoMO individuals may have learned from past interactions that engaging in similar (even immoral) behaviors as valued others commit can influence resource allocation. Thus, such violations may be a proactive approach to ensuring they maintain or gain social resources.

### Fear of Missing Out

**Fig 7:**
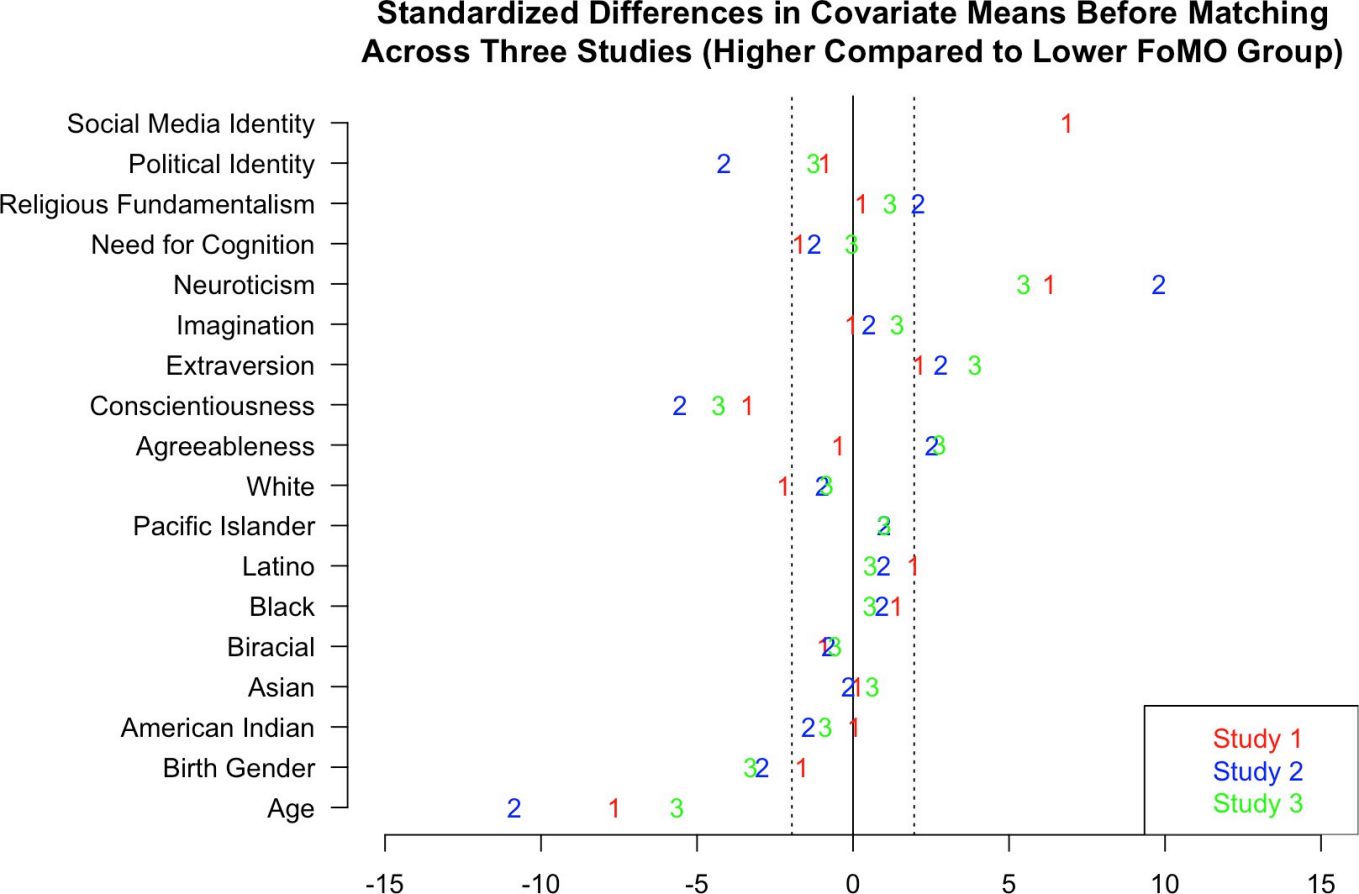
Baseline differences in higher and lower FoMO groups as shown via standardized differences in covariate means before matching.

We found evidence that there may be some important personality, demographic, and cognitive differences between those with higher and lower FoMO (see Fig 7 below). Just considering those variables that were found to have baseline differences in all three of the studies, we found four. Those with higher FoMO have increased neuroticism and extraversion but are younger and with lower conscientiousness compared to lower FoMO peers. The potential differences increase when you consider those variables repeatedly different in two of the three studies. These potentially include higher FoMO individuals being more agreeable and more likely male, which aligns with past research [1].

### Limitations, considerations, and future directions

Our choice of a median split for matching and comparison was to be as conservative as possible while maintaining an appropriate sample size for all analyses. Importantly, however, we did not just rely on comparing FoMO as a binary variable with median split using Fisher Tests but also examined FoMO as a continuous variable using GAM. Thus, these two separate approaches provided convergent and agreeing evidence that such relationships exist and are robust to such choices in analysis, even though dichotomous splits on continuous variables by themselves may not always be ideal. Future research can use different comparison methods to see whether these associations found here hold constant or increase in magnitude.

Another important consideration is the possibility of additional influences not being accounted for in this work. Because this is the first study series to assess the relationship between FoMO and moral cognition using causal modeling, we likely did not account for all variables involved in moral awareness, judgment, and behaviors. This study provides an important avenue for future work as all behavior is multiply determined. Still, we used a strong and conservative approach via RCM which held potentially relevant covariates static among conditions to rule out many potential confounds. Yet it is possible that influential variables were not included in our analysis, and so future work should focus on identifying other potential factors related to FoMO and moral cognition. For instance, FoMO represents a form of anxiety, so it is important to rule out other forms of anxiety (general anxiety) or traits (need to belong) that might alter how individuals process social information.

Additionally, future research should build on this work by considering how various states might influence the relationship between FoMO and moral judgments. For example, some work finds that sleep issues can result in a negative interpretive bias to increase hostile workplace interpretations regarding unfair and ambiguous treatment [22], so sleep issues may also influence morally relevant social information judgments. Thus, ample room exists to increase our understanding of how various traits and states affect the cognitive processing of morally related social information. However, it is worth noting that recent work claims that philosophical intuitions (i.e., moral judgments) are relatively stable and unaffected by attempts to manipulate state variables [60].

Lastly, although we did not preregister these studies or complete *a priori* power analyses, the observed effects regarding moral judgment were consistent and reliably detected across three studies. As discussed above, the results regarding moral awareness were more equivocal, suggesting the possible presence of moderating variables. Yet, as this was the first series of studies to examine these relationships, little information (i.e., observed effect sizes) was available to inform power analysis. Therefore, we collected large samples and used targeted approaches to match individuals in those samples when conducting RCM analyses. Some may question specifically whether the sample size of Study 1 was sufficient for propensity score matching procedures. However, research indicates that propensity score matching is effective even in samples smaller than the one used for Study 1 [61]. Still, this approach provides some information useful for future researchers in determining power *a priori* as it provides initial effect estimates useful for that approach.

## Conclusion

We conducted three studies examining how FoMO influences moral awareness, judgments, and recall/prediction of past/future moral violations. These three studies confirmed that increased FoMO is associated with less severe moral judgments, increased recall of past and higher expectations of future moral transgressions regarding the self and one’s social network. The influence of FoMO on moral awareness was more ambiguous, providing additional opportunities for future research. We hope the present findings provide impetus for future work delineating the influences of FoMO on individuals’ moral awareness, judgments, and behavior.

## Supporting information

S1 Text Vignette validation and non-linear GAM results. This file contains additional information for the vignette validation and non-linear plots for GAMs.
(DOCX)
