# Supplementary material for: Fear of Missing Out’s (FoMO) relationship with moral judgment and behavior
Source: PLoS One. 2024 Nov 7;19(11):e0312724. doi: 10.1371/journal.pone.0312724 (PMC11542806; doi:10.1371/journal.pone.0312724)

**Vignettes**

**Moral Vignettes**

Theft I

Social Condition. All of your closest friends have bought tickets to see a music

artist’s last concert before they retire. These have been your best friends since you were a kid. You haven’t got paid from work this week and don’t have enough money to buy a ticket. You ask your significant other if they will buy you the ticket. Your significant other says no because they are saving for new clothes. You see their wallet left on the table and buy the last ticket with their money so you can attend the concert with your best friends.

Non-social Condition. A website is selling tickets to see a music artist’s last concert before they retire. You haven’t got paid from work this week and don’t have enough money to buy a ticket. You ask your significant other if they will buy you the ticket. Your significant other says no because they are saving for new clothes. You see their wallet left on the table and buy the last ticket with their money so you can attend the concert.

Theft II

Social Condition. All of your closest friends bought tickets to the midnight premiere of a movie. These have been your best friends since you were a kid. You ask your parents if they could give you the money to buy the movie ticket. Your parents say no because it is a school night. Your parents leave their wallet open in their room and you take the money anyways so you can watch the movie with your best friends.

Non-social Condition. A website is selling tickets to the midnight premiere of a movie. You ask your parents if they could give you the money to buy the movie ticket. Your parents say no because it is a school night. Your parents leave their wallet open in their room and you take the money anyways so you can watch the movie.

Vandalism/destruction of property

Social Condition. You have a test the day after mischief night, but all of your closest friends invite you to participate. These have been your best friends since you were a kid. They have already purchased eggs, spray paint, and toilet paper. You put on all black clothes and a mask so you cannot be identified and head out with your friends.

Non-social Condition. You have a test the day after mischief night. You have already purchased eggs, spray paint, and toilet paper. You put on all black clothes and a mask so you cannot be identified and head out by yourself.

Giving away Rx drugs

Social Condition. You’re on a plane going to Italy for spring break with your best friends. These have been your best friends since you were a kid. One of your close

friends admits they have anxiety and they do not do well on planes. You offer to give your prescription Xanax. Your friend accepts and you give them Xanax for the flight there.

Non-social Condition. You’re on a plane going to Italy for spring break. The stranger sitting next to you admits they have anxiety and they do not do well on planes. You offer to give your prescription Xanax away. They accept and you give them Xanax for the flight there.

Sexual assault

Social Condition. Your best friend is throwing a house party while their parents are away. As the night progresses, you see the person you have been talking to drunkenly stumble up to your best friend’s room to pass out. All of your closest friends have already lost their virginities. These have been your best friends since you were a kid. You leave for college tomorrow and don’t want to go as a virgin. Your best friends come up to you and encourage you to take this opportunity. You make sure no one besides your friends see you head up to the room to go and have sex with this person while they’re passed out.

Non-social Condition. A kid is throwing a house party while their parents are away. As the night progresses, you see the person you have been talking to drunkenly stumble up to the host’s room to pass out. You leave for college tomorrow and don’t want to go as a virgin. You make sure no one sees you head up to the room to go and have sex with this person while they’re passed out.

Lying/dishonesty

Social Condition. All of your closest friends decided on a last-minute day trip to go wine tasting at the local vineyards. These have been your best friends since you were a kid. You are scheduled to work and are unable to find anyone to cover your shift. You know that this is the last time that one of your best friends will be there before they move to a different state. You call into work and lie about being sick so you can go spend the day wine tasting with your friends.

Non-social Condition. You decided on a last-minute day trip to go wine tasting at the local vineyards. You are scheduled to work and are unable to find anyone to cover your shift. You know that this is the last time that these vineyards will be open for the season. You call into work and lie about being sick so you can go spend the day wine tasting.

Infidelity

Social Condition. You are at a club with your best friends where your favorite music artist is performing. These have been your best friends since you were a kid. After the show, the artist comes up to you and ask if you’re single and if you want to go back to their hotel room with them for an after party. You lie about not being in a relationship knowing that this is a once in a lifetime opportunity to hook up with a celebrity and get your best friends into the after party.

Non-social Condition. You are at a club where your favorite music artist is performing.

After the show, the artist comes up to you and ask if you’re single and if you want to go back to

their hotel room with them for an after party. You lie about not being in a relationship knowing that this is a once in a lifetime opportunity to hook up with a celebrity.

Reckless driving/endangering others

Social Condition. You’re working on the night of the super bowl. You were supposed to get out in time to watch it with all of your closest friends, but your relief is running late. These have been your best friends since you were a kid. By the time they finally arrive, it’s almost half time. You go 100mph on the highway, so you don’t miss the halftime show with your friends.

Non-social Condition. You’re working on the night of the super bowl. You were supposed to get out in time to watch it, but your relief is running late. By the time they finally arrive, it’s almost half time. You go 100mph on the highway, so you don’t miss the halftime show.

Substance abuse

Social Condition. You recently started working at a small family owned restaurant with your best friends. These have been your best friends since you were a kid. Although they do not drug test, the company has a strict no drug policy. You start getting high before your shifts with your best friends.

Non-social Condition. You recently started working at a small family owned restaurant. Although they do not drug test, the company has a strict no drug policy. You start getting high before your shifts.

Laziness/sloth

Social Condition. Your parent’s friend just got you a job working security at a store they own. You are not allowed to be on your phone, sleep, or read books during your shift. Your duties are to watch the cameras and walk around the store every hour. You sit in your own little room by yourself where no one can see you. You spend your entire shift on your phone keeping up with all the pictures your best friends are posting on social media for their monthly friends get together. These have been your best friends since you were a kid.

Non-social Condition. Your parent’s friend just got you a job working security at a store they own. You are not allowed to be on your phone, sleep, or read books during your shift. Your duties are to watch the cameras and walk around the store every hour. You sit in your own little room by yourself where no one can see you. You spend your entire shift on your phone keeping up with all the pictures National Geographic is posting on social media for their monthly recap.

Physical harm

Social Condition. A local store is the exclusive vendor for concert tickets that all your closest friends already have tickets to. These have been your best friends since you were a kid. You turn into the parking lot and see that there is already a large crowd surrounding the entrance waiting for the store to open. The tickets are limited, and you aren’t sure how many there are left. You do know that isn’t enough for everyone in line. You push through the crowd, elbowing and knocking some people over so that you can be the first one in the store to get a ticket to go to the concert with your friends.

Non-social Condition. A local store is the exclusive vendor for concert tickets. You turn into the parking lot and see that there is already a large crowd surrounding the entrance waiting for the store to open. The tickets are limited, and you aren’t sure how many there are left. You do know that isn’t enough for everyone in line. You push through the crowd, elbowing and knocking some people over so that you can be the first one in the store to get a ticket to go to the concert.

Ingratitude

Social Condition. You work the night shift as a nurse at the local hospital. You have tonight off and can’t wait to go see a midnight movie premier with your closest friends. These have been your best friends since you were a kid. Russel, a coworker, texts you and asks if you can take his shift for the night so he can celebrate his anniversary. Even though he has switched last minute with you the past four times you asked him to, you ignore the text and go watch the movie with your friends.

Non-social Condition. You work the night shift as a nurse at the local hospital. You have tonight off and can’t wait to go see a midnight movie premier. Russel, a coworker, texts you and asks if you can take his shift for the night so he can celebrate his anniversary. Even though he has switched last minute with you the past four times you asked him to, you ignore the text and go watch the movie.

Impoliteness/impatience

Social Condition. It is a beautiful Saturday morning and you have plans to hang out with your best friends at the beach. These have been your best friends since you were a kid. As you get onto the highway you see that traffic is all backed up from everyone trying to head out and enjoy their day. You only needed to get on the highway to get off at the next exit. You don’t feel like waiting behind all these other people that are also trying to get off the exit because you have fun plans. You get over and ride the shoulder the next half mile to pass everyone so you can get off the exit and hang out with your friends at the beach.

Non-social Condition. It is a beautiful Saturday morning and you have plans to hang out at the beach. You get onto the highway you see that traffic is all backed up from everyone trying to head out and enjoy their day. You only needed to get on the highway to get off at the next exit. You don’t feel like waiting behind all these other people that are also trying to get off the exit because you have fun plans. You get over and ride the shoulder the next half mile to pass everyone so you can get off the exit and hang out at the beach.

Selling drugs

Social Condition. You live in a large apartment building. You find out from your closest friends that your new next-door neighbor has an addiction to painkillers. These have been your best friends since you were a kid. You remember you have some left over Oxycodone pills from your arm surgery 2 months ago. You were originally planning on just flushing them down the toilet, but you decide to sell them to your neighbor so you can afford to go out to the bar with your best friends.

Non-social Condition. You live in a large apartment building. You find out that your new next-door neighbor has an addiction to painkillers. You remember you have some left over Oxycodone pills from your arm surgery 2 months ago. You were originally planning on just flushing them down the toilet, but you decide to sell them to your neighbor so you can afford to go out to the bar.

**Vignette Validation**

Here we wanted to validate and explore the vignettes used in the studies. We tested the following preregistered hypotheses:

H1) The acts in the moral violation vignettes will be judged as wrong compared to neutral or not wrong.

H2) The acts in the moral violation vignettes will be thought of as having

people in general society judging it as wrong compared to neutral or not wrong.

H3) The acts in the moral violation vignettes will be seen as ensuring that

agents would not miss out on a rewarding experience that others were having if they did

not engage in such behavior.

**Method**

***Participants***

We recruited an approximately nationally representative panel of participants through Qualtrics. All participants gave informed consent. Data was collected online. Institutional Review Board (IRB) approval was abstained from Duke University. An a priori power analysis was conducted using G*Power (Faul et al, 2007) to determine the minimum sample size required to test the study hypotheses. Results indicated that the required sample size to achieve 80% power for detecting an effect of 0.3 at a significance criterion of a = .05 was N = 352 for the difference between two independent means. We recruited 404 participants to account for bad or missing data.

***Procedures and Materials***

Participants were randomly assigned to one of four study conditions: Moral Social, Moral Non-social, Non-moral Social, and Non-moral non-social. Participants saw all 14 vignettes for their respective condition and for each vignette, judged how wrong the action was, selected the domain that best describes the behavior, reported what percentage of people in general society would believe such action is at least somewhat [morally] wrong, and then reported the chance that if a person did not engage in such a behavior that they would miss out on a rewarding experience that others were having. Although we collected information on non-moral vignettes, as the examination of non-moral judgments is not examined or reported in this study, we will only discuss the moral violation vignettes here.

***Measures***

**Vignettes.** Vignettes are the same as used in the other studies.

***Statistical Analysis.***

To test the respective hypotheses in R, one-sample and independent-sample t-tests were run. All data and code are available here: <https://osf.io/7d6yz/>.

**Results**

***Hypothesis 1: Judgments of Acts in Moral Vignettes***

For acts depicted in the moral vignettes, participants judged them as morally wrong (*M* = -38.33, *SD* = 52.48, *95% CI* [-45.63, -31.04], *t*(200) = -10.357, *p* < .001), supporting H1.

***Hypothesis 2: Perceived Judgments by the General Society***

Participants believed that the general society would judge acts in the moral vignettes as morally wrong (*M* = 66.60, *SD* = 19.65, *95% CI* [63.87, 69.34], *t*(200) = 48.048, *p* < .001), corroborating H2.

***Hypothesis 3: Missing Out on Rewarding Experiences***

Regarding the notion of agents missing out on rewarding experiences if they did not engage in the depicted behavior, acts in the moral vignettes were seen as ensuring engagement (*M* = 53.50, *SD* = 26.41, *95% CI* [49.83, 57.18], *t*(200) = 28.718, *p* < .001), validating H3.

**Discussion**

This study aimed to validate the moral vignettes used in our studies. Our findings suggest they were appropriate for investigating the relationship between FoMO and moral judgments.

In line with our first hypothesis (H1), participants judged the moral vignettes as significantly morally wrong, suggesting a clear cognitive distinction from neutral or not-wrong acts.

The second hypothesis (H2) evaluated participants' perceptions of how the general society would judge the acts in the vignettes. Findings showed that participants believed that society, as a whole, would also judge these acts as morally wrong. This highlights a perceived societal consensus on these moral judgments, emphasizing their utility in these studies' context.

Our third hypothesis (H3) touched upon the Fear of Missing Out (FoMO) regarding rewarding experiences if certain questionable behaviors were not engaged in as described in the vignettes. Moral vignettes evoked a significant FoMO response, suggesting that they were appropriate for use in these studies’ context.

In conclusion, our findings indicate that the vignettes used in these studies were appropriate to answer the primary questions of interest.

**GAM Non-Linear Results**

**Study 1 Results**

**Moral Awareness**


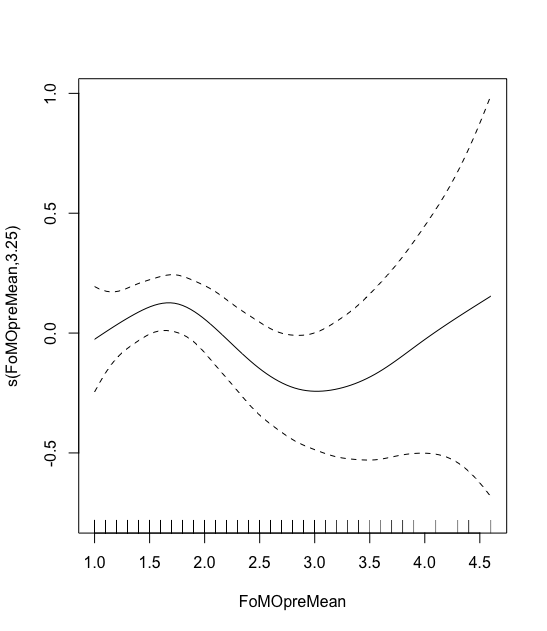

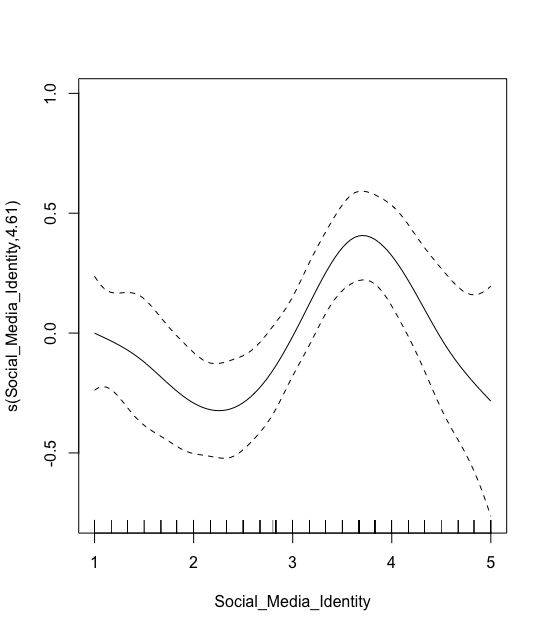


**Moral Judgment**


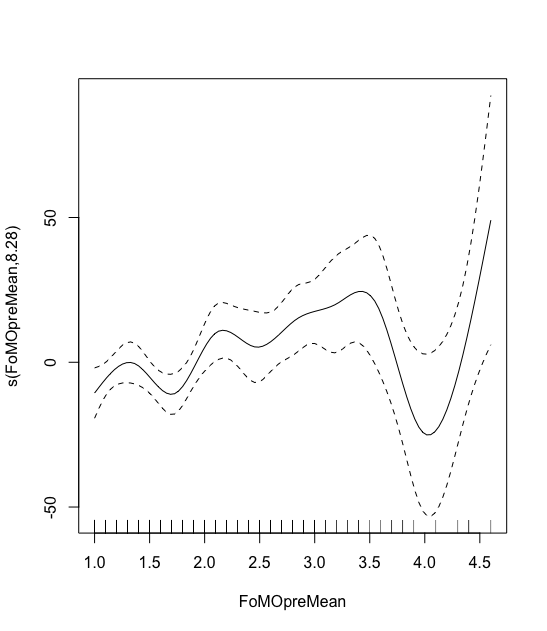

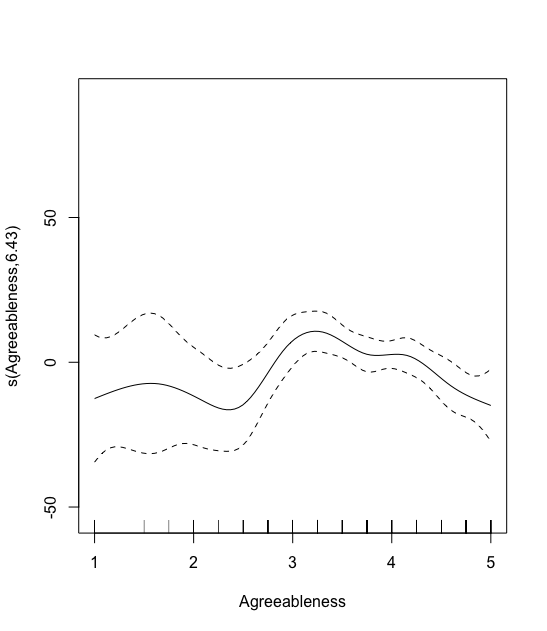


**Personal Recall**


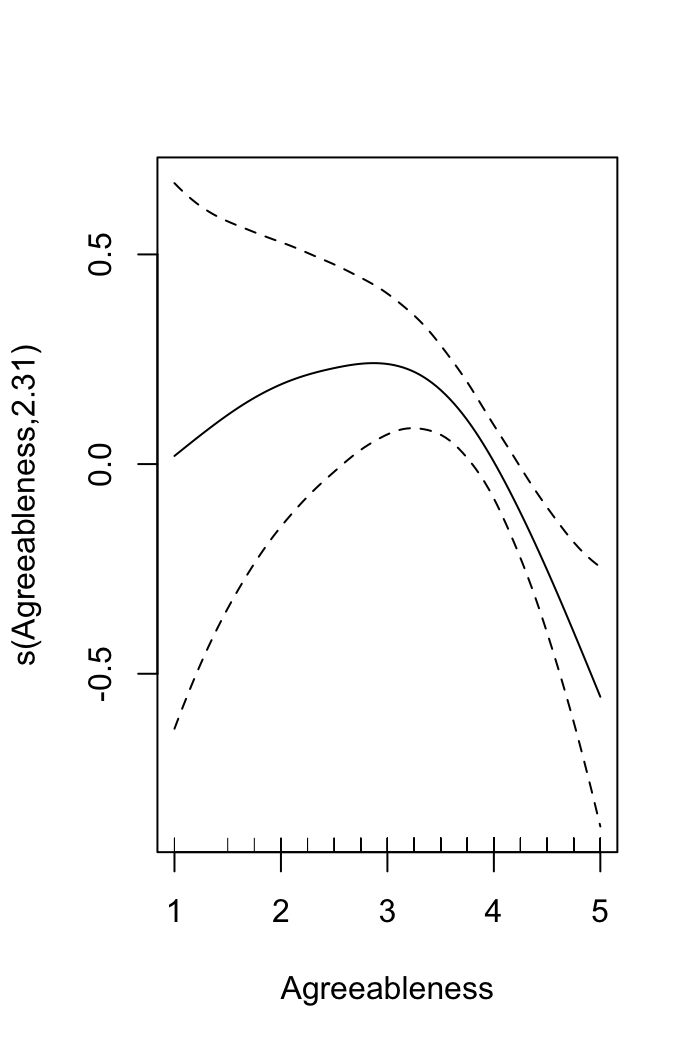


**Social Recall**


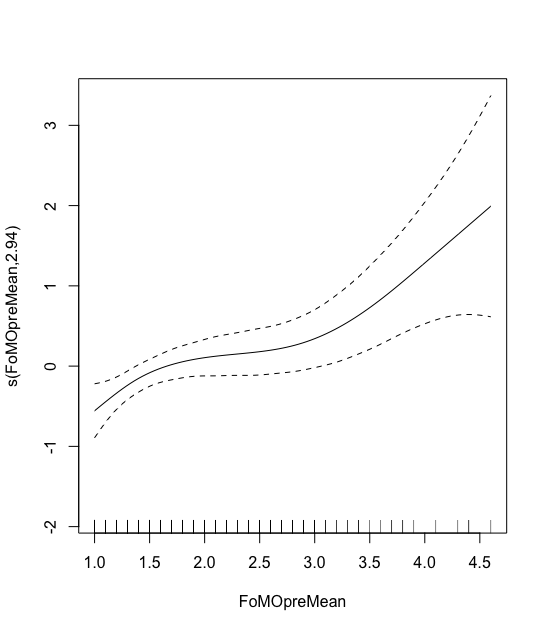

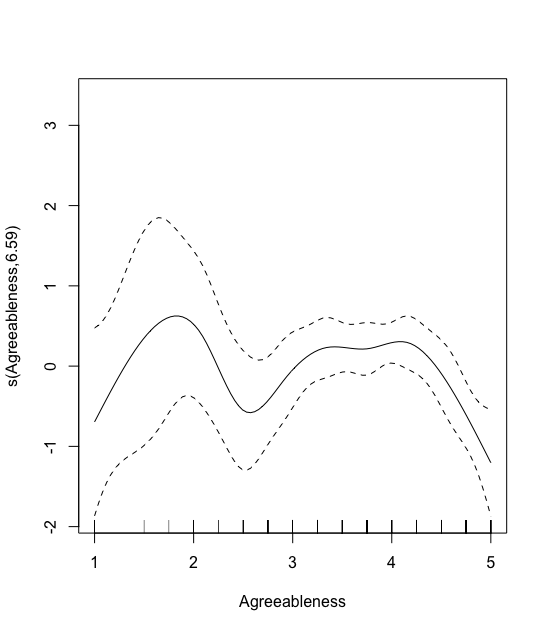


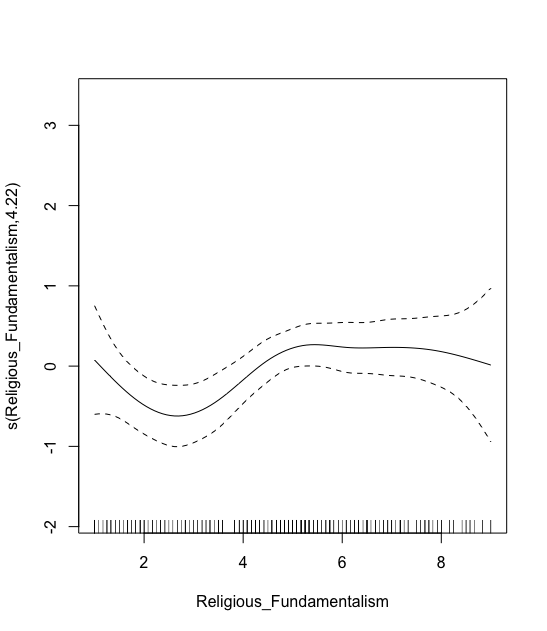


**Personal Prediction**


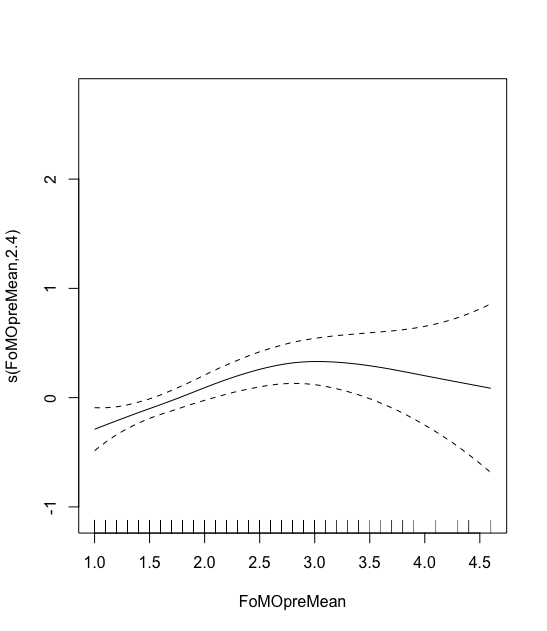

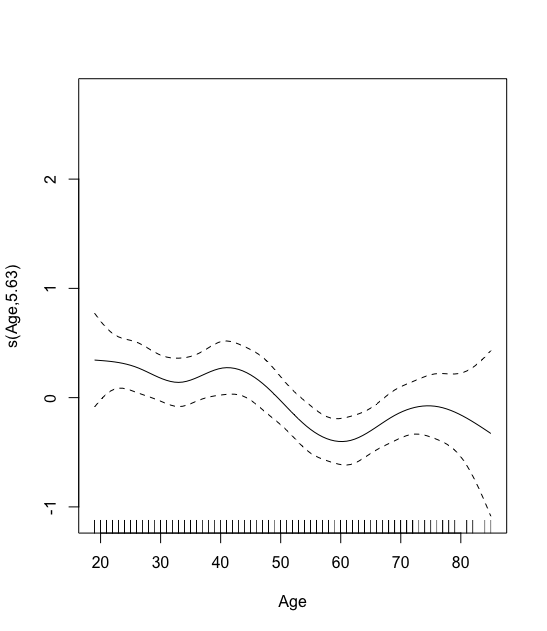


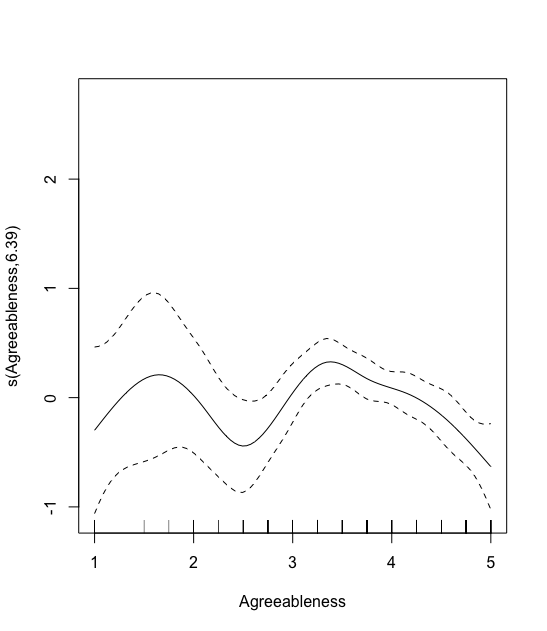

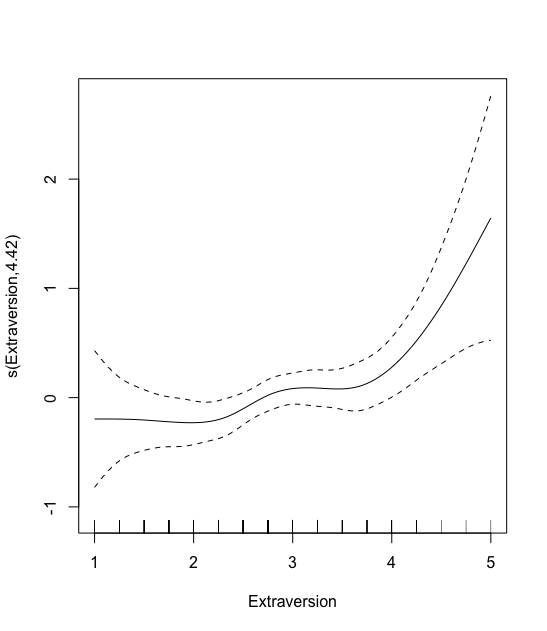


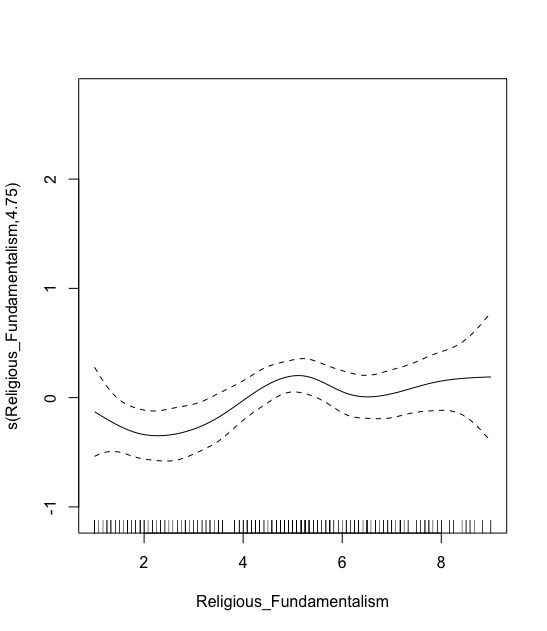


**Social Prediction**


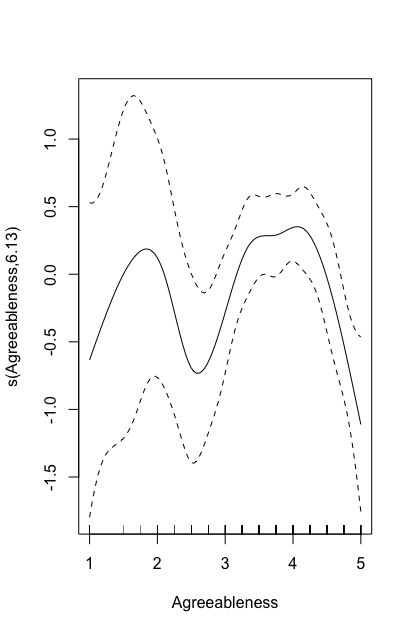

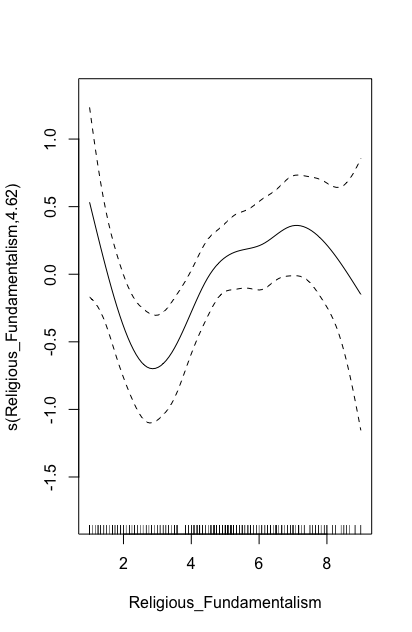


**Study 2 Results**

**Moral Awareness**


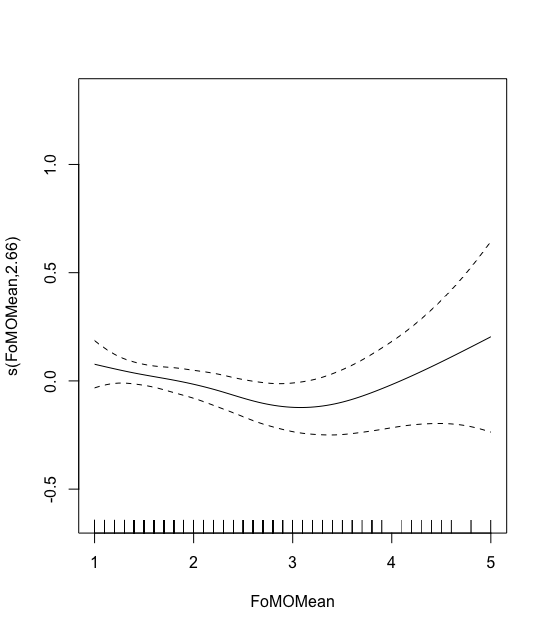

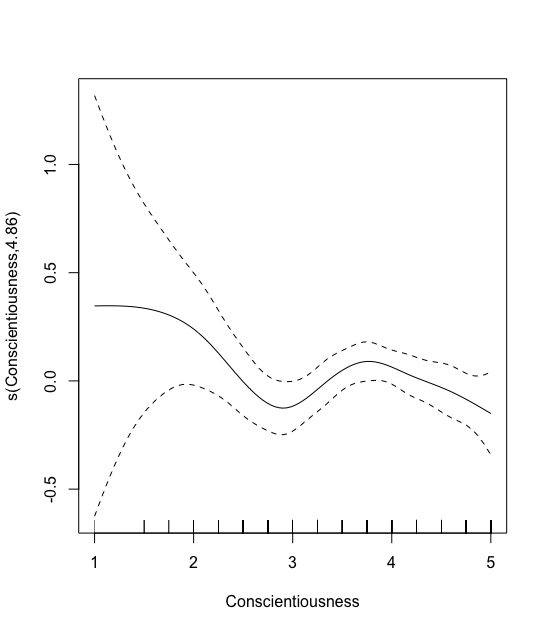


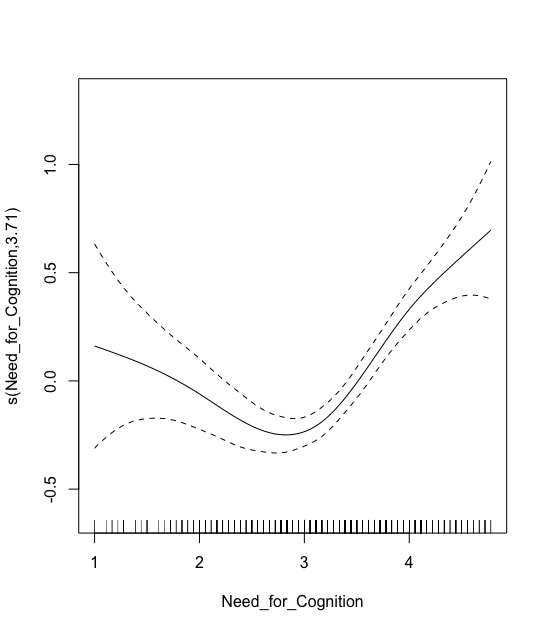

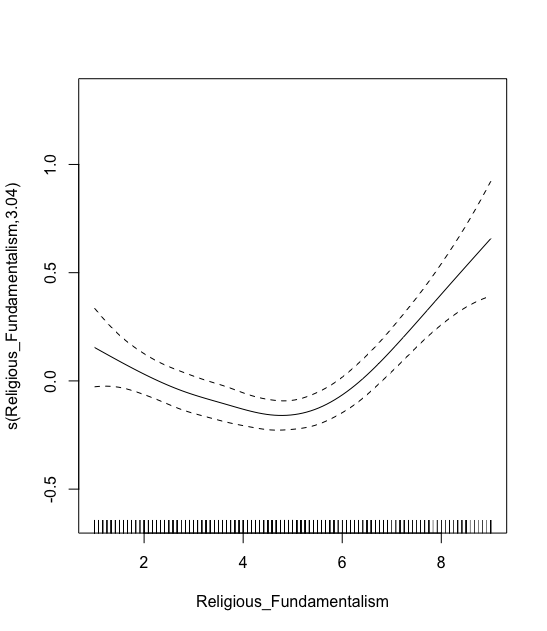


**Moral Judgment**


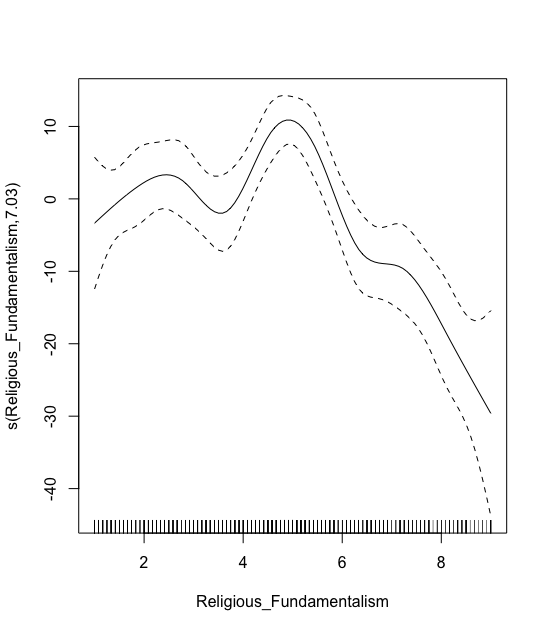


**Personal Recall**


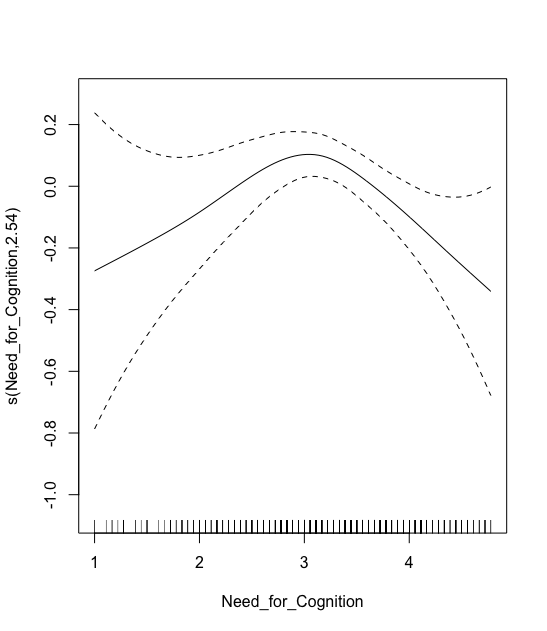

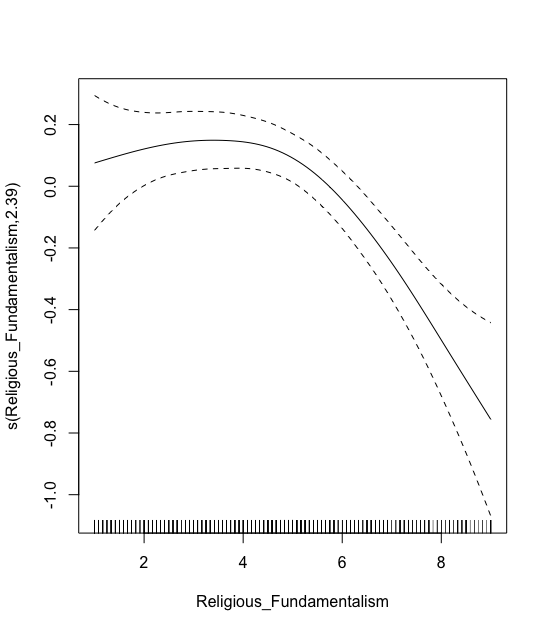


**Social Recall**


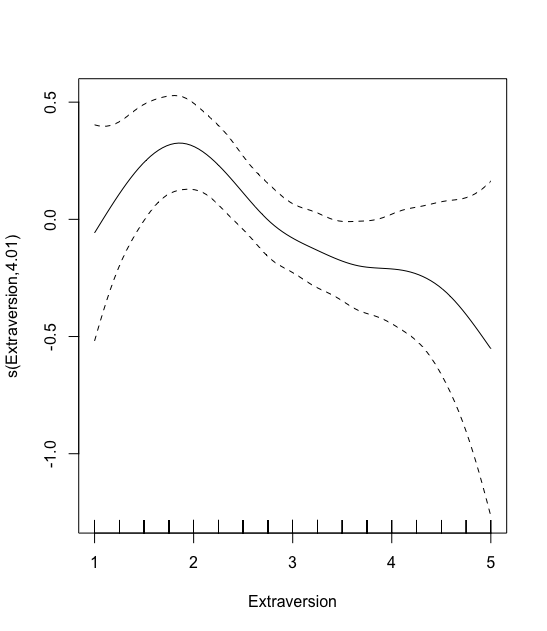


**Personal Prediction**


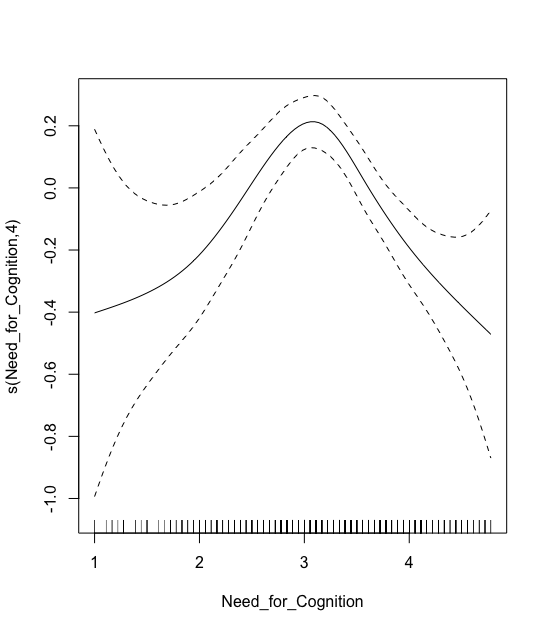

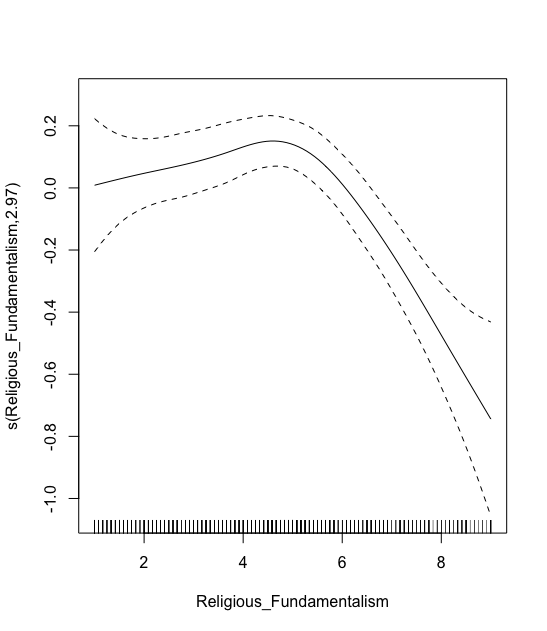


**Social Prediction**


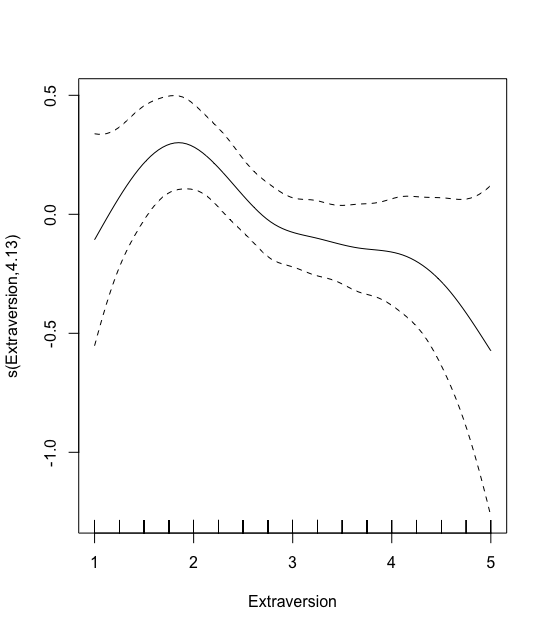


**Study 3 Results**

**Moral Awareness**


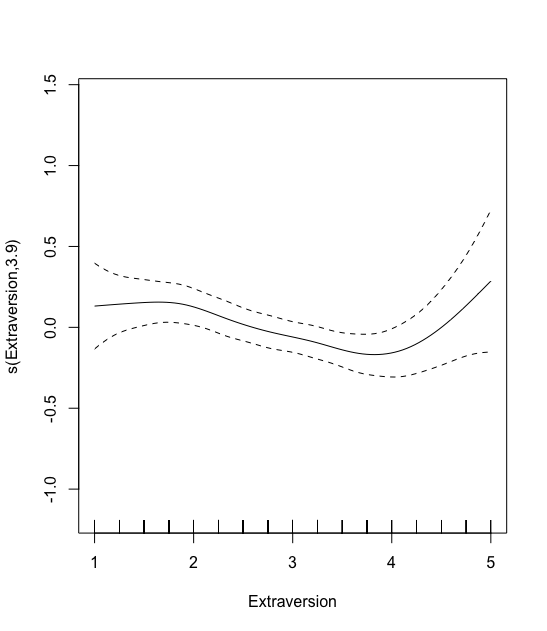

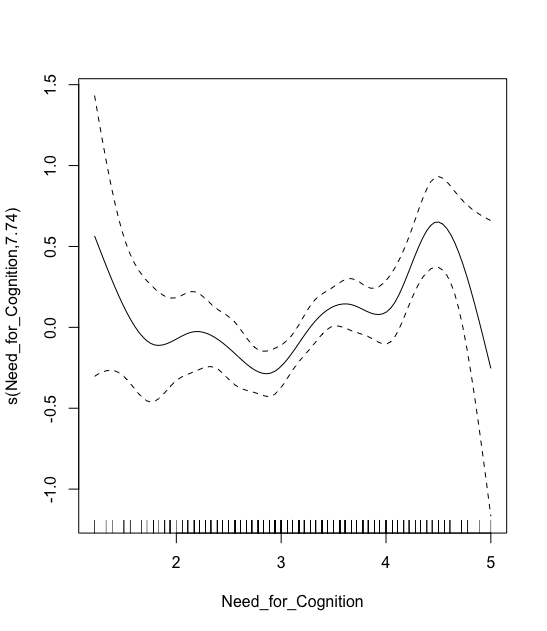


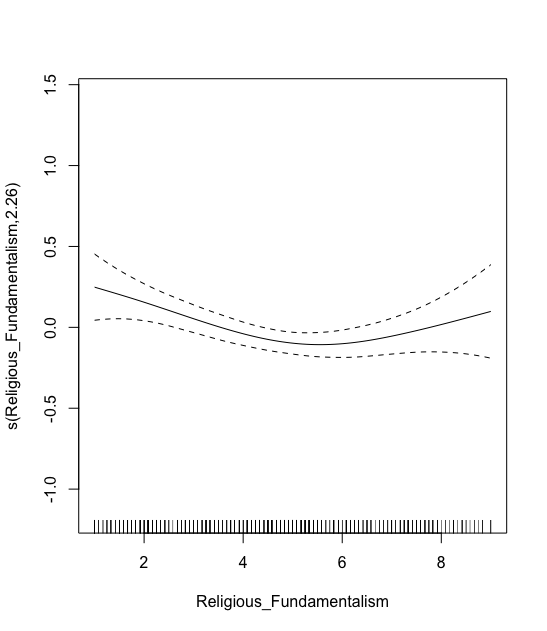


**Moral Judgment**


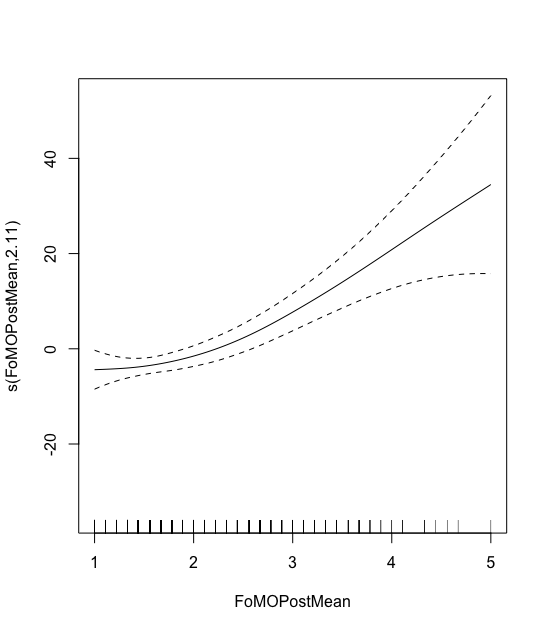

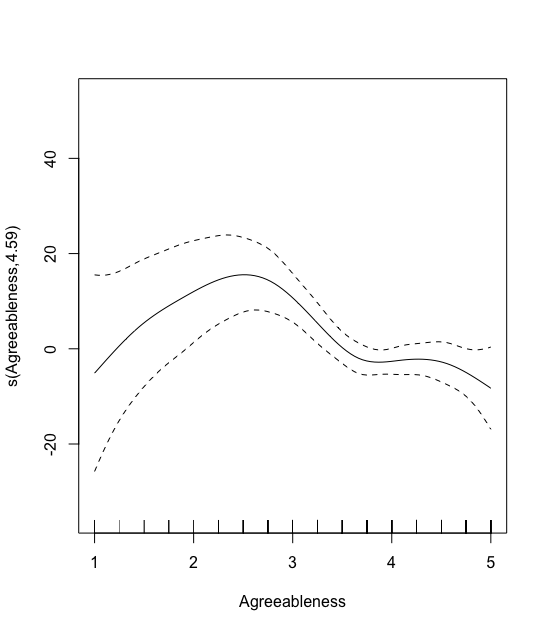


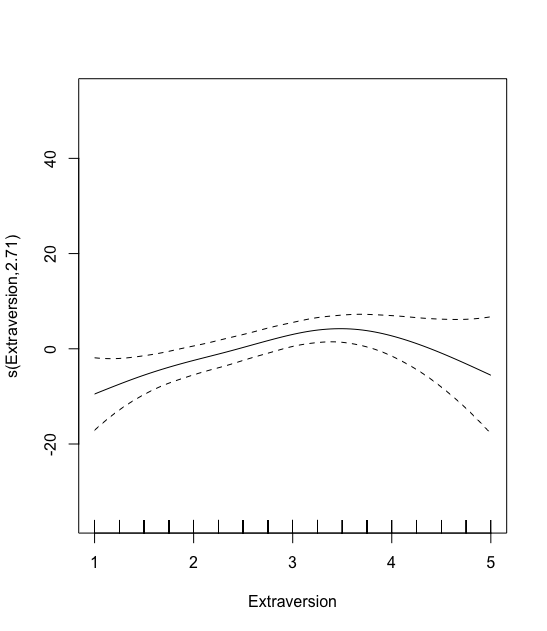

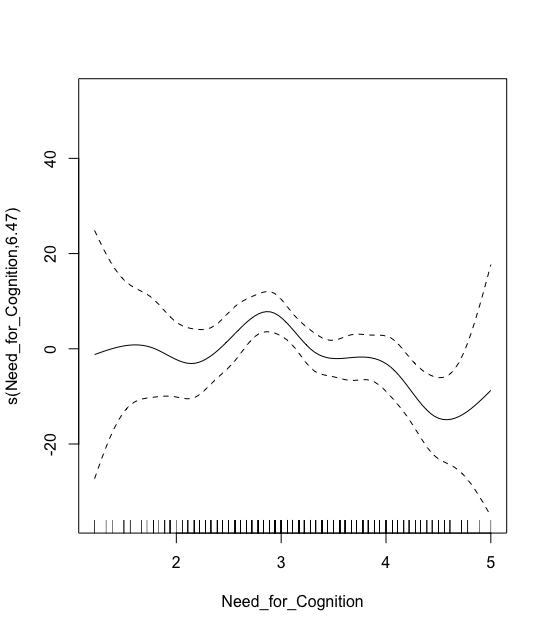


**Personal Recall**


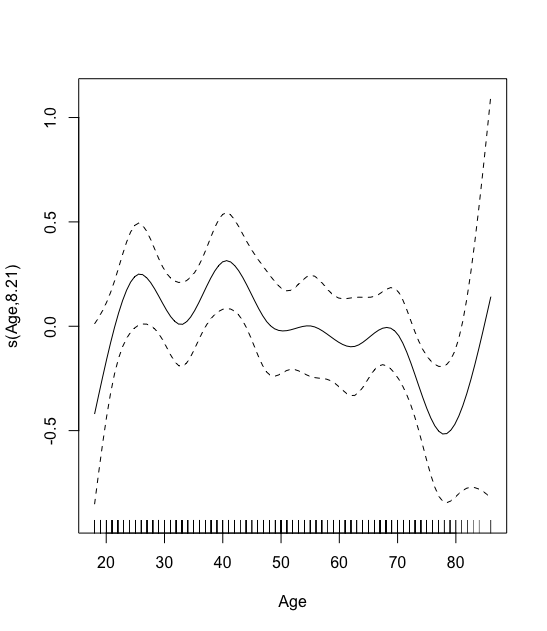

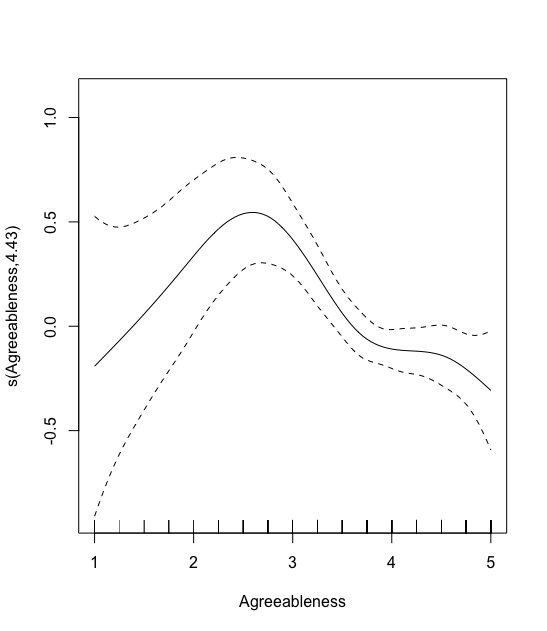


**Social Recall**


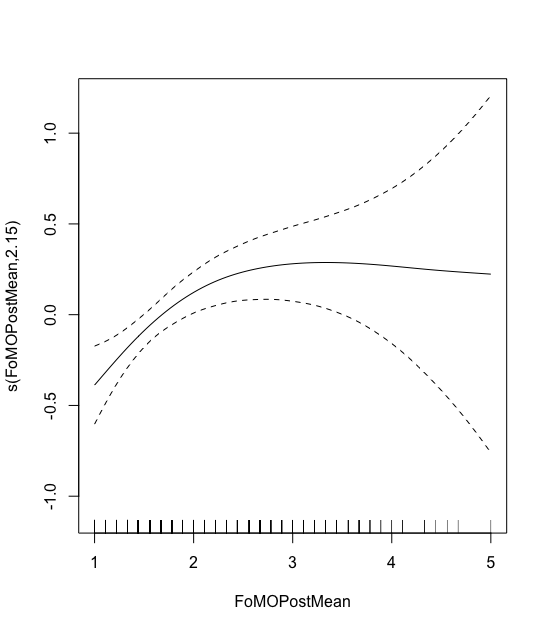

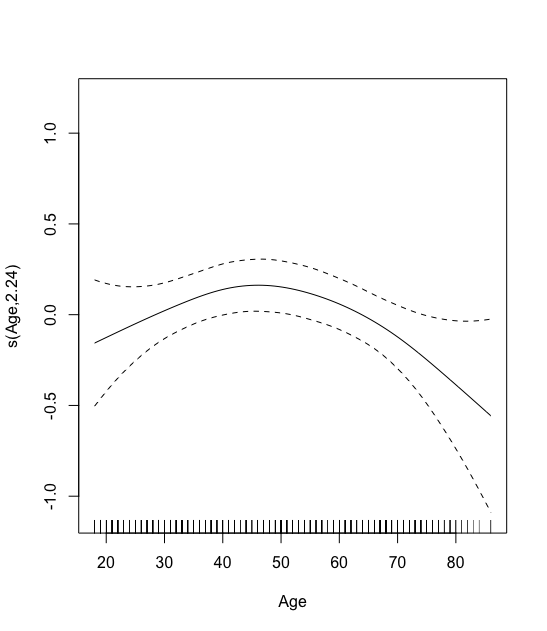


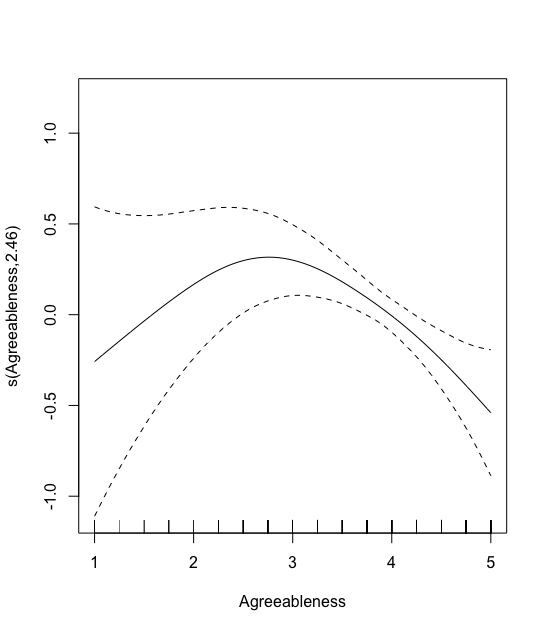

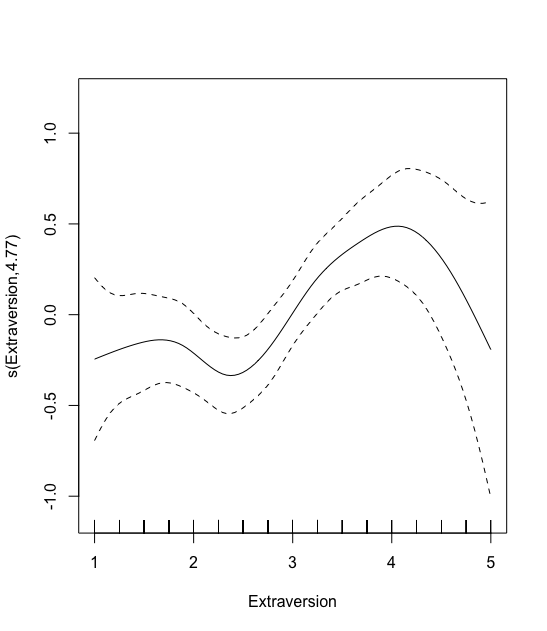


**Personal Prediction**


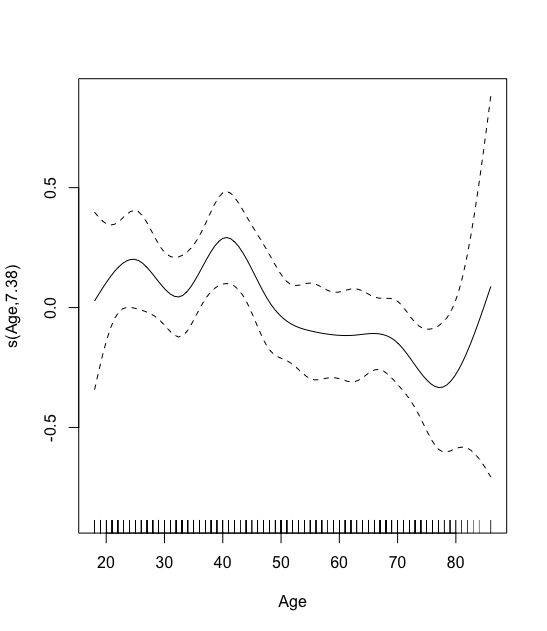

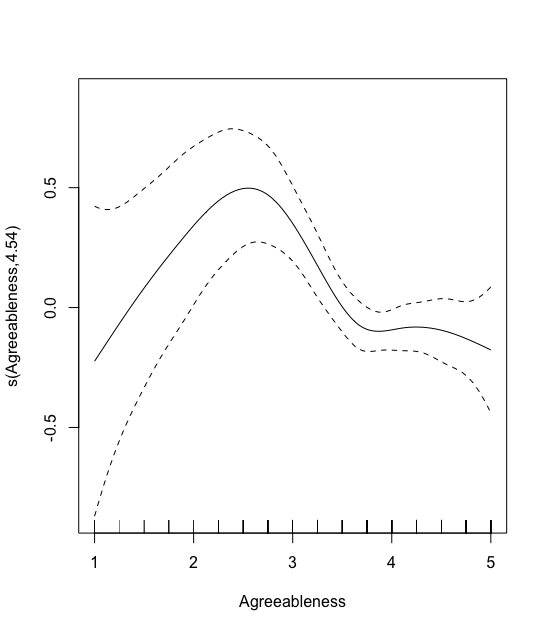


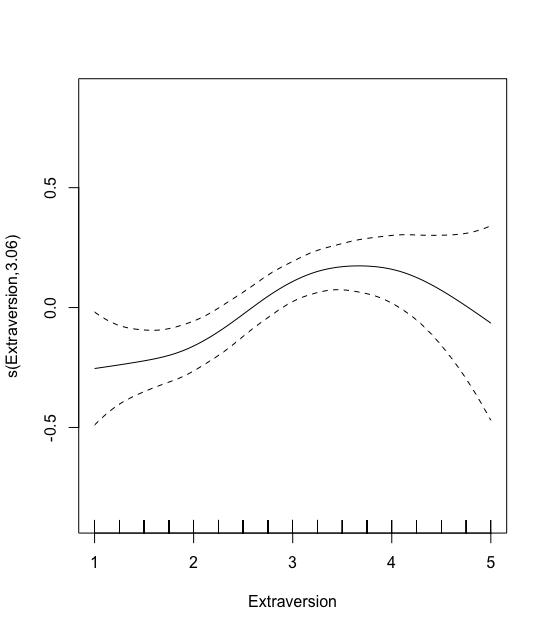


**Social Prediction**


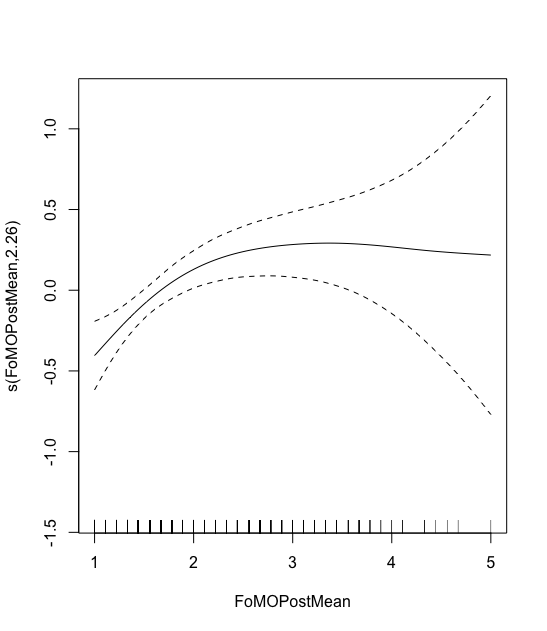

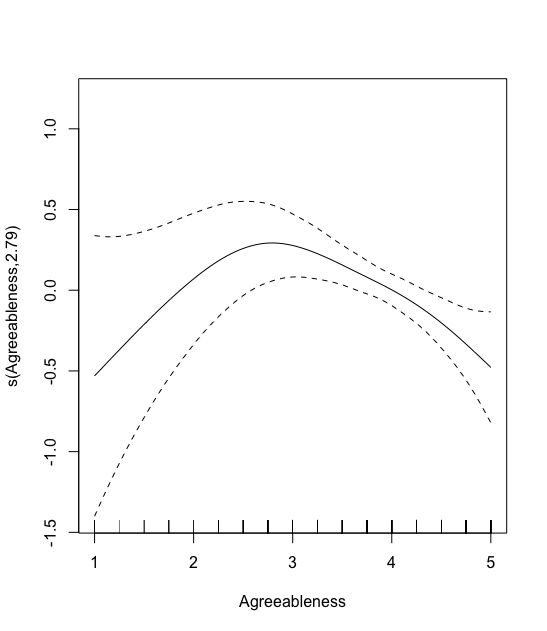


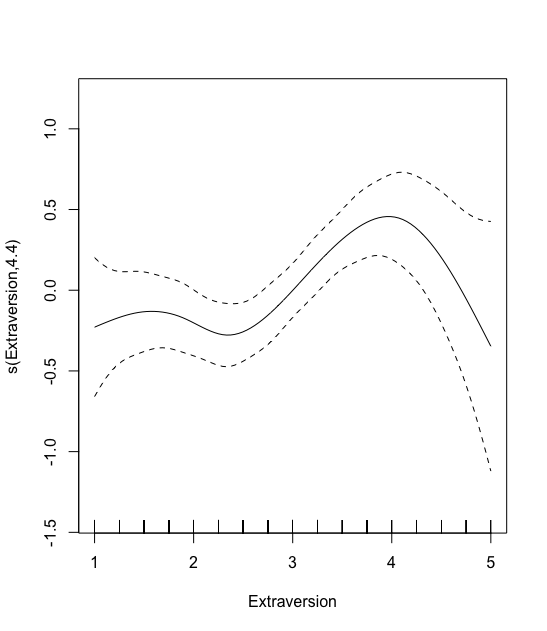

Supplement: S1 Text — This file contains additional information for the vignette validation and non-linear plots for GAMs. (DOCX) [file pone.0312724.s001.docx]
